# Discovery of novel serum metabolic biomarkers in patients with polycystic ovarian syndrome and premature ovarian failure

**DOI:** 10.1080/21655979.2021.1982312

**Published:** 2021-10-26

**Authors:** Jiying Chen, Qinger Zhou, Yonggang Zhang, Wenqing Tan, Hanchao Gao, Liying Zhou, Shuixiu Xiao, Jinhua Gao, Jing Li, Zhiying Zhu

**Affiliations:** a Department of Obstetrics and Gynecology, Shenzhen Longhua District Central Hospital, Guangdong Medical University Affiliated Longhua District Central Hospital, Shenzhen, China; b Department of Clinical Laboratory, Shenzhen Longhua District Central Hospital, Guangdong Medical University, Shenzhen, China; c Department of General Practice, Shenzhen Longhua District Central Hospital, Guangdong Medical University Affiliated Longhua District Central Hospital, Shenzhen, China; d Department of Medical Laboratory, Shenzhen Longhua District Central Hospital, Guangdong Medical University Affiliated Longhua District Central Hospital, Shenzhen, China

**Keywords:** metabolic biomarkers, polycystic ovarian syndrome, premature ovarian failure

## Abstract

Several widely recognized metabolites play a role in regulating the pathophysiological processes of various disorders. Nonetheless, the lack of effective biomarkers for the early diagnosis of polycystic ovarian syndrome (PCOS) and premature ovarian failure (POF) has led to the discovery of serum-based metabolic biomarkers for these disorders. We aimed to identify various differentially expressed metabolites (DEMs) through serum-based metabolic profiling in patients with PCOS and POF and in healthy individuals by using liquid chromatography–mass spectrometry analysis. Furthermore, heatmap clustering, correlation, and Z-score analyses were performed to identify the top DEMs. Kyoto Encyclopedia of Genes and Genomes enriched pathways of DEMs were determined using metabolite-based databases. Moreover, the clinical significance of these DEMs was evaluated on the basis of area under the receiver operating characteristic curve. Significantly dysregulated expressions of several metabolites were observed in the intergroup comparisons of the PCOS, POF, and healthy control groups. Furthermore, 6 DEMs were most frequently observed among the three groups. The expressions of these DEMs were not only directly correlated but also exhibited potential significance in patients with PCOS and POF. Novel metabolites with up/downregulated expressions can be discovered in patients with PCOS and POF using serum-based metabolomics; these metabolites show good diagnostic performance and can act as effective biomarkers for the early detection of PCOS and POF. Furthermore, these metabolites might be involved in the pathophysiological mechanisms of PCOS and POF via interplay with corresponding genes.

## Introduction

Polycystic ovary syndrome (PCOS) is a common endocrine disorder that is generally diagnosed in women of childbearing age [1]. PCOS is characterized by the presence of small, round cysts in the ovary; however, it is much more complex than the mere presence of cysts [2]. PCOS-related metabolic irregularities include anovulation, infertility, hyperandrogenism, insulin resistance, hyperinsulinemia, and abnormal hair growth [3]. Long-term PCOS increases the risk of type 2 diabetes, cardiovascular disease, and metabolic syndrome [4]. Patients with PCOS require regular reproductive support; nevertheless, during gestation, they are at risk of developing complications that might compromise fetal outcomes, such as pre-eclampsia and gestational diabetes [4,5]. Early diagnosis of PCOS is challenging because of its variable nature and the different diagnostic criteria [6].

Premature ovarian failure (POF), also known as premature ovarian insufficiency, is a condition characterized by loss of ovarian function, premature follicular depletion or absence of menarche, and cessation of manstruation and folliculogenesis before the age of 40 years [7]. Metabolic disorders such as galactosemia; autoimmune adrenal and thyroid diseases; genetic factors such as chromosomal abnormalities; infectious diseases such as mumps; oxidative stress; iatrogenic factors such as chemotherapy and radiotherapy; type 2 diabetes; and ovarian granulosa cell apoptosis are commonly implicated in the pathophysiology of POF [8–10].

Detection of circulating biomarkers can facilitate the screening of cancer or other diseases, comprehension of disease biology, and early detection of recurrence accompanied by minimum invasion [11]. Recently, circulating biomarker-based studies have gained tremendous attention because of the discovery of many serum-based molecules, such as miRNAs, metabolites, and proteins [12]. Liquid chromatography–mass spectrometry (LC–MS) is a powerful tool with various applications, and LC–MS-based metabolomics analyses have been performed to identify circulating biomarkers for multiple disorders [13–15]. Different studies have been conducted to determine single or multiple biomarkers derived from tissue samples or body fluids, such as serum, plasma, urine, and saliva, that can be utilized to detect and diagnose diseases [16,17]. Several metabolic biomarkers might be implicated in the etiology of PCOS and POF, playing crucial roles in the occurrence and progression of these diseases [18,19].

Various metabolites have been widely recognized and found to play a role in regulating the pathophysiological processes of various disorders. Nonetheless, the lack of effective biomarkers for early diagnosis of PCOS and POF has led to the discovery of serum-based metabolic biomarkers in patients with PCOS and POF.

## Materials and methods

### Patients

Serum samples of a total of 100 participants (patients with PCOS, n = 31; patients with POF, n = 43; and healthy controls, n = 26) visiting the Shenzhen Longhua District Central Hospital, Guangdong Medical University Affiliated Longhua District Central Hospital, between May 2020 and April 2021 were retrospectively collected. All participants signed informed consent during their hospital stay. Study approval was obtained from the ethics committee of the Shenzhen Longhua District Central Hospital, Guangdong Medical University Affiliated Longhua District Central Hospital, following which the study was conducted in accordance with the Declaration of Helsinki.

### Collection and pre-treatment of serum samples

Serum samples were collected, stored at −80°C, and thawed at 4°C for subsequent analysis. Each sample (100 µL) was transferred into 2 mL centrifuge tubes, following which 400 µL methanol (−20°C) was added and the mixture vortexed for 60 s. Afterward, the sample tubes were centrifuged at 12,000 rpm for 10 min at 4°C, and the supernatant was transferred to new centrifuge tubes. The samples were concentrated to dryness in a vacuum. Following this, the samples were dissolved in 150 µL 2-chlorobenzalanine (4 ppm) methanol (80%) solution, and the supernatant was filtered through a 0.22 µm membrane to obtain the initial samples for LC–MS analysis. Before the analysis, 20 µL of each sample was used for quality control (QC) [20–22].

### LC–MS analysis

Chromatographic separation was performed using Waters ACQUITY UPLC HSS T3 (150 × 2.1 mm, 1.8 µm Waters Corporation, Massachusetts, USA) columns of Thermo Vanquish system (Thermo Fisher Scientific Inc., Massachusetts, USA) maintained at 40°C. Gradient elution of analytes was performed using 5 mM ammonium formate in water (A3) and acetonitrile (B3) or 0.1% formic acid in water (A2) and acetonitrile (B2) at a flow rate of 0.25 mL/min. After equilibration, 2 µL of each sample was injected. The linear gradient of solvent (B2/B3) (v/v) was used as follows: 2% B2/B3 (0 to 1 min); 2% B2/B3 (1 to 9 min); 2% to 50% B2/B3 (9 to 12 min); 50% to 98% B2/B3 (12 to 13.5 min); 98% B2/B3 (13.5 to 14 min); 98% to 2% B2/B3 (14 to 20 min); 2% B2-positive model (14 to 17 min); and 2% B3-negetive model (14 to 17 min) [20–23].

Electrospray ionization (ESI)–tandem mass spectrometry analyses were performed using the Thermo Q Exactive HF-X mass spectrometer (Thermo Fisher Scientific Inc., Massachusetts, USA) with a spray voltage of 3.5 kV in positive mode and −2.5 kV in negative mode. The capillary temperature was 325°C. The analyzer was scanned over a mass range of m/z 81–1000 for a full scan with a mass resolution of 60,000. Data-dependent acquisition MS/MS analyses were performed via higher energy collisional dissociation scan. Dynamic exclusion was performed in the MS/MS spectra to remove unnecessary information [20–23].

### Data analysis

R (version 3.3; Boston, MA, USA) and SPSS (20.0 version; SPSS Inc., Chicago, IL, USA) software were used for all bioinformatics and statistical analyses, and data were expressed as mean ± standard deviation. Bioinformatics tools were used to analyze clustering heatmaps, bar plots, correlation matrix, and Kyoto Encyclopedia of Genes and Genomes (KEGG) enrichment pathways related to the top DEMs. The top DEMs were identified using fold change, p values, variable importance in projection scores, and one-way and two-way analysis of variance (ANOVA)-based t-tests. One-way ANOVA was performed for between-group or multiple-group comparisons. Pearson’s correlation was used for correlation analysis. The clinical significance of the top DEMs in serum samples was evaluated using the area under the receiver operating characteristic (ROC) curve (AUC). Significance was set at P < 0.05.

## Results

The study aims to discover noval serum metabolic biomarkers by LC–MS-based metabolomics, and provide clinical significance in patients with PCOS, POF and heathy controls. Herein, we retrospectively evulated 100 participants of three groups and carried out hypothesis.

### Profiling of metabolites

**Figure 1.**
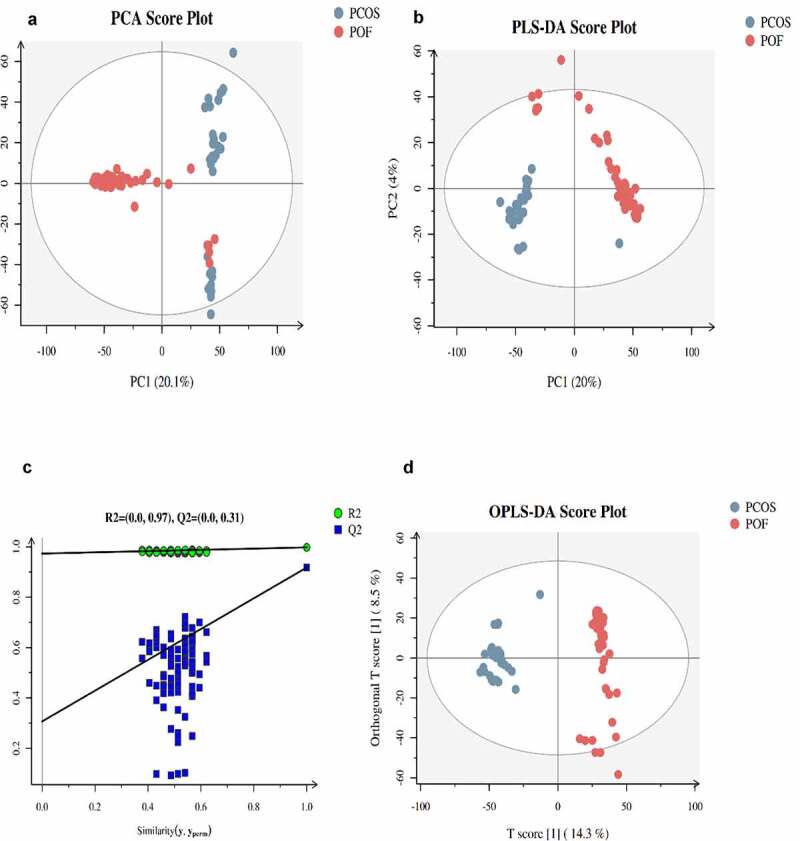
**Score plots of three different analyses based on ESI^+^ mode in PCOS and POF**. (a) Principal component analysis (PCA), (b,c) Partial least discriminant analysis (PLS-DA) (d) Orthogonal partial least square discriminant analysis (OPLS-DA) was used for displaying PCOS and POF subjects

**Figure 2.**
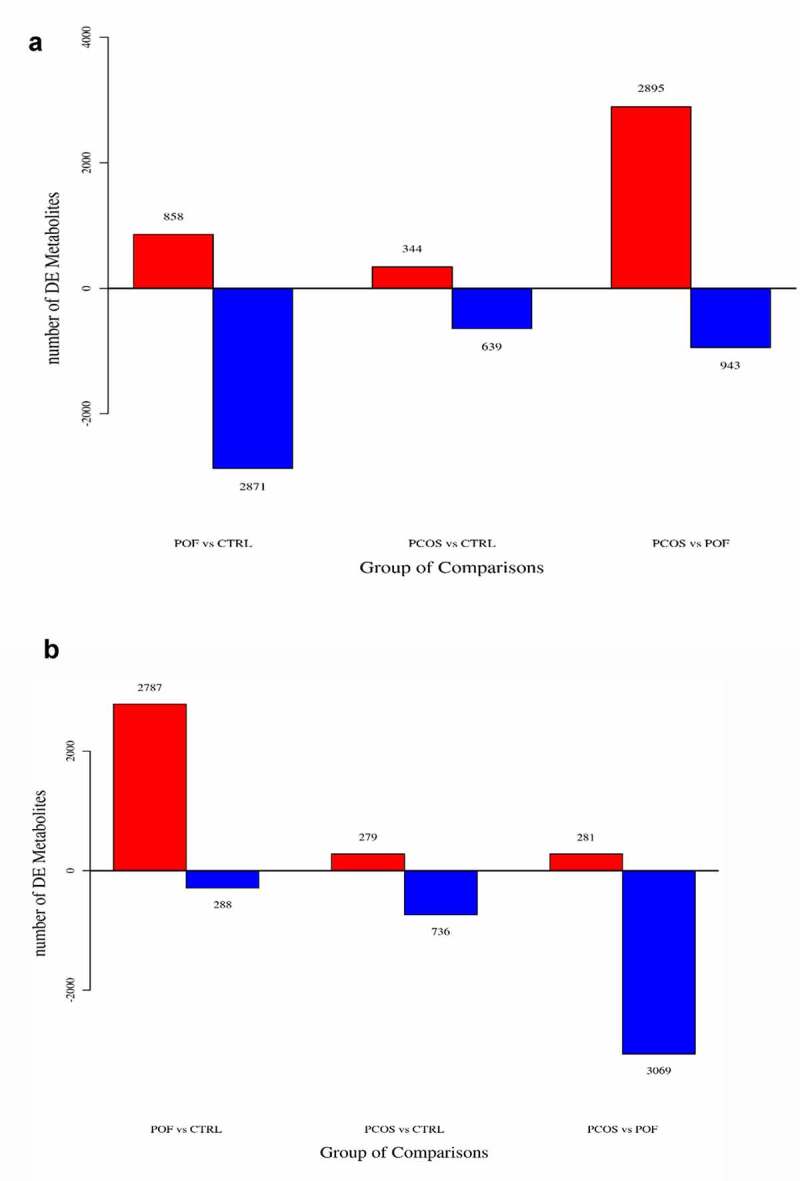
**The identification of total DEMs in between three groups**. Bar plots represent the total expressed DEMs in between ESI^+^ (a) ESI^−^ (b) modes

The general extracted ion chromatograms from the two ESI (positive and negative) modes are shown in the base peak chromatogram in **Supplementary Figure S1 (A,B)**. Additionally, the QC samples in positive and negative modes were clustered together in the principal component analysis (PCA) score plots that verified the quality of the samples (**Supplementary Figure S2 (A,B)**). Relative standard deviation peaks with a coefficient of variation showed that data obtained from QC samples were robust and reproducible, as shown in **Supplementary Figure S2 (C,D)**. Moreover, three different methods of multivariate analysis, ie, PCA, partial least squares discriminant analysis, and orthogonal partial least squares discriminant analysis, were used to identify the top significant DEMs among the PCOS, POF, and healthy control (CTRL) groups on the basis of certain threshold criteria, such as 1)p-value ≤ 0.05 and variable importance in projection (VIP) ≥ 1; 2)p-value ≤ 0.05 and fold_change ≥ 1.5 or ≤0.667; 3) p-value ≤ 0.05 (Multiple Groups) (Figure 1(a-d)**, Supplementary Figure S3**). Overall, 983, 3729, and 3838 DEMs were identified in PCOS vs CTRL, POF vs CTRL, and PCOS vs POF group metabolites, respectively, in positive mode (Figure 2a), whereas 1015, 3075, and 3350 DEMs, respectively, were identified in negative mode (Figure 2b) using matched annotations of an in-house developed database (Panomic) and online databases including Human Metabolome Database, METLIN, LipidMaps, and mzCloud.

### PCOS vs CTRL metabolites

**Figure 3.**
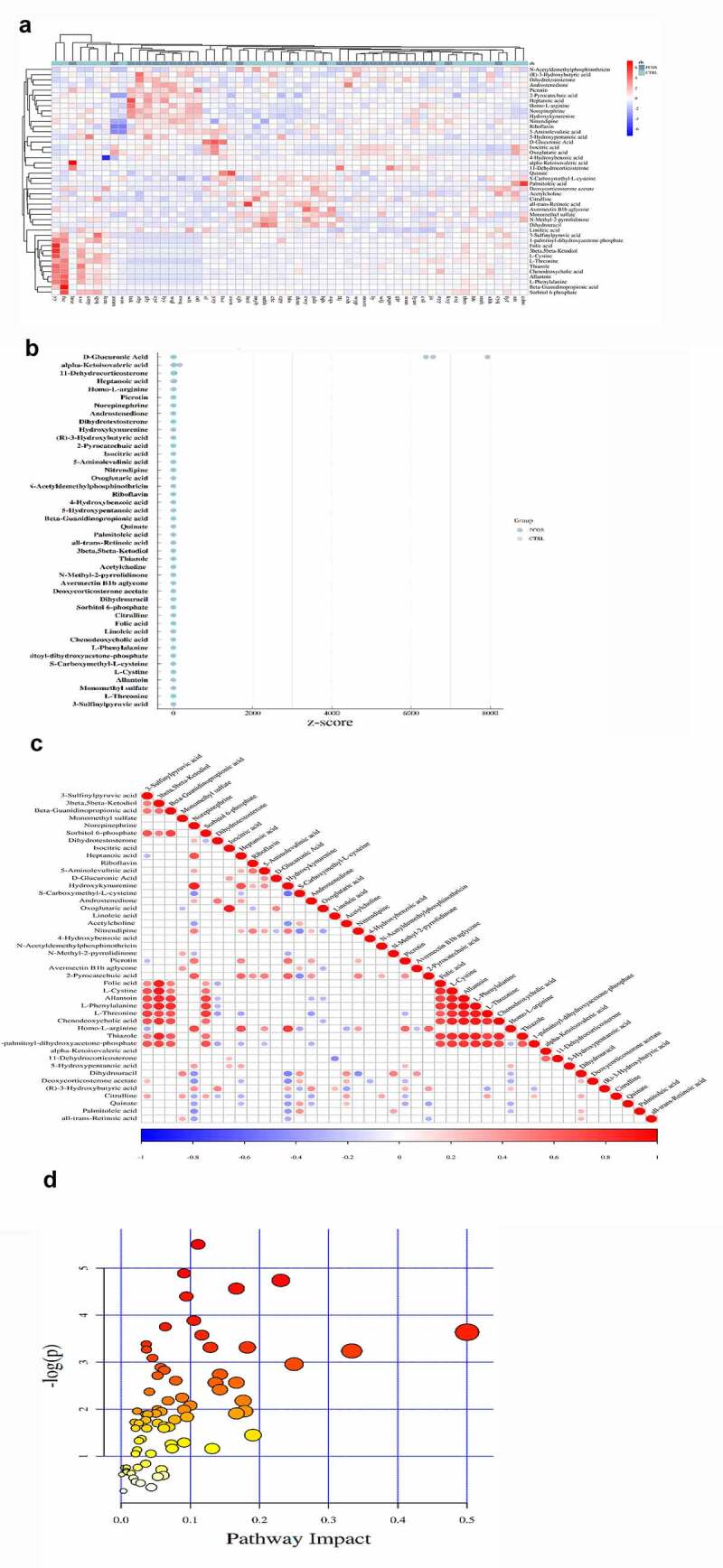
**The significant DEMs were discovered and verified in PCOS vs CTRL group**. (a) Heatmap clustering represented the significant DEMs. (b) Z score statistical analysis was done for obtaining high-rank DEMs. (c) Pearson correlation coefficient analysis was performed between each DEM. (d) KEGG pathways related to metabolites were presented via bubble plot. Bigger the bubble, higher the involved significant metabolites

The top 10 significant DEMs identified in the PCOS vs CTRL group comprised those with upregulated expressions including D-glucuronic acid, alpha-ketoisovaleric acid, 11-dehydrocorticosterone, hepatonic acid, and picrotin and those with downregulated expressions including beta-guanidinopropionic acid, L-cystine, all-trans retinoic acid, folic acid, and 3beta,5beta-ketodiol (Table 1). Moreover, these DEMs were identified via clustering heatmap (Figure 3a) and further analyzed using Z-score (Figure 3b); additionally, positive and negative correlations were determined using correlation coefficient analysis (Figure 3c). Furthermore, the top 5 significantly enriched KEGG pathways for PCOS vs. CTRL group metabolites included cyclic adenosine monophosphate signaling pathways; cancer pathways, such as those in prostate cancer cells; synaptic vesicle cycle pathway; central carbon metabolism pathways in cancer; and protein digestion and secretion pathways. These pathways were identified via KEGG pathway-based MetPA tool (Figure 3d and Table 2).

**Table 1.** Top-ranked DEMs discovered by metabolomics in between three groups

	VIP	FC	Log2 FC	P value	FDR
***PCOS vs CTRL***					
D-Glucuronic Acid	1.313	435.63	8.767	0.019	0.27
Alpha-Ketoisovaleric acid	1.019	8.979	3.167	0.01	0.242
11-Dehydrocorticosterone	2.076	6.405	2.679	0.01	0.242
Heptanoic acid	1.937	2.867	1.52	0.015	0.238
Picrotin	2.097	1.904	0.929	0.044	0.41
Beta- Guanidinopropionic acid	1.575	0.158	−2.664	0.001	0.086
L-Cystine	2.604	0.189	−2.402	5.79E-05	0.058
All-trans-Retinoic acid	1.603	0.224	−2.156	0.045	0.347
Folic acid	2.348	0.266	−1.909	5.41E-05	0.058
3beta,5beta-Ketodiol	2.006	0.272	−1.88	0.001	0.075
***POF vs CTRL***					
18-Hydroxycorticosterone	1.294	7.372	2.882	1.41E-06	4.3E-06
2-Arachidonoylglycerol	1.228	6.845	2.775	9.78E-08	4.15E-07
Rimantadine	1.213	4.049	2.018	3E-08	1.5E-07
Pentostatin	1.999	4.018	2.006	8.18E-06	0.000151
Mirtazapine	1.563	3.724	1.897	2.34E-07	8.84E-07
Taurocyamine	1.216	0.016	−5.931	3.34E-10	4.58E-09
L-4-Hydroxyphenylglycine	1.843	0.034	−4.883	8.86E-13	1.35E-10
Hydroquinone	1.257	0.048	−4.38	2.1E-10	3.25E-09
Retinol	2.036	0.067	−3.909	5.17E-12	3.39E-10
Dicyclomine	1.644	0.077	−3.708	1.06E-10	2.11E-09
***PCOS vs POF***					
L-4-Hydroxyphenylglycine	1.565	23.08	4.529	1.22E-12	1.6E-10
Dicyclomine	1.007	15.353	3.941	8.83E-09	4.78E-08
Uracil 5-carboxylate	1.492	12.544	3.649	5.59E-09	3.3E-08
myo-Inositol	1.316	11.188	3.484	8.63E-12	3.64E-10
Retinol	1.714	10.774	3.429	7.2E-12	3.33E-10
Ethylmethylacetic acid	1.427	0.065	−3.935	6.19E-09	3.79E-07
2-Arachidonoylglycerol	1.255	0.159	−2.651	1.28E-09	9.96E-09
D-Fructose	1.274	0.181	−2.464	5.59E-09	3.3E-08
18-Hydroxycorticosterone	1.295	0.183	−2.449	2.1E-06	5.96E-06
Gamma-L-Glutamyl-L-2-aminobutyrate	1.103	0.187	−2.419	4.16E-07	1.39E-06

Abbreviation: VIP, variable important on projection; FC, Foldchange, FDR, false-discovery rate.

**Table 2.** Top-ranked KEGG pathways obtained related to DEMs metabolites in between three groups

**Pathways**	**Pathway** **Codes**	**Total** **Metabolites**	**Differential Metabolites**	**Impact** **Scores**	**P value**
***PCOS vs CTRL***					
cAMP signaling pathway	hsa04024	25	3	0.11111	0.0040728
Pathways in cancer	hsa05200	31	3	0.090909	0.0075314
Prostate cancer	hsa05215	11	2	0.23077	0.0087473
Synaptic vesicle cycle	hsa04721	12	2	0.16667	0.010409
Central carbon metabolism in cancer	hsa05230	37	3	0.09434	0.01232
Salivary secretion	hsa04970	17	2	0.10526	0.020568
Protein digestion and absorption	hsa04974	47	3	0.06383	0.02346
Intestinal immune network for IgA production	hsa04672	2	1	0.5	0.026326
Citrate cycle (TCA cycle)	hsa00020	20	2	0.11667	0.028023
ABC transporters	hsa02010	138	5	0.11111	0.034032
***POF vs CTRL***					
ABC transporters	hsa02010	138	19	0.13768	3.00E-05
Protein digestion and absorption	hsa04974	47	10	0.21277	6.59E-05
Central carbon metabolism in cancer	hsa05230	37	8	0.16981	0.00032311
Mineral absorption	hsa04978	29	7	0.2	0.00037662
Cortisol synthesis and secretion	hsa04927	12	4	0.33333	0.0020315
Cushing syndrome	hsa04934	13	4	0.31579	0.0028231
Arginine and proline metabolism	hsa00330	78	10	0.38725	0.004285
Valine, leucine and isoleucine biosynthesis	hsa00290	23	5	0.29032	0.0043849
GABAergic synapse	hsa04727	9	3	0.41176	0.0078337
Glutathione metabolism	hsa00480	38	6	0.18531	0.0095182
***PCOS vs POF***					
Protein digestion and absorption	hsa04974	47	15	0.31915	1.01E-08
ABC transporters	hsa02010	138	24	0.17391	2.07E-07
Central carbon metabolism in cancer	hsa05230	37	11	0.24528	2.30E-06
Aminoacyl-tRNA biosynthesis	hsa00970	52	11	0.21429	7.89E-05
Prostate cancer	hsa05215	11	5	0.46154	0.00016079
Pathways in cancer	hsa05200	31	8	0.24242	0.00017828
Ovarian steroidogenesis	hsa04913	24	7	0.29545	0.00019958
Mineral absorption	hsa04978	29	7	0.2	0.00071327
GABAergic synapse	hsa04727	9	4	0.64706	0.00086673
Taste transduction	hsa04742	32	7	0.23684	0.0013384

### POF vs CTRL metabolites

**Figure 4.**
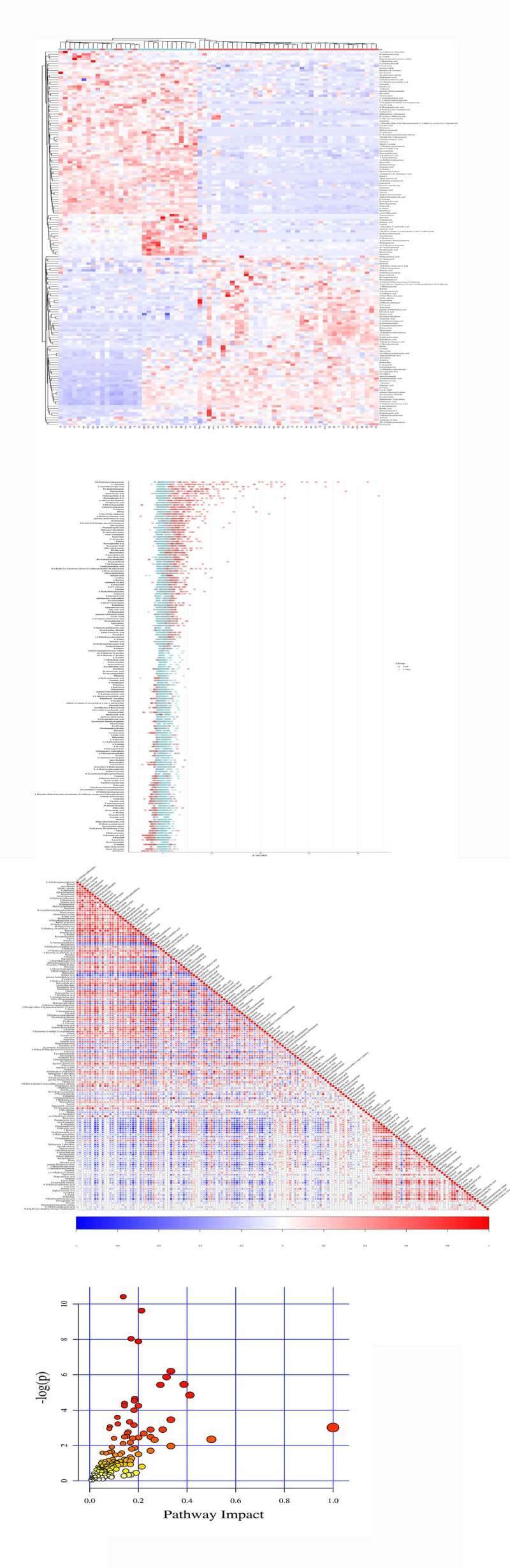
**The significant DEMs were discovered and verified similarly in POF vs CTRL group**. (a) Heatmap clustering showed significant DEMs. (b) Z score statistical analysis was performed for obtaining high-rank DEMs. (c) Pearson correlation coefficient analysis was done between each DEM. (d) KEGG pathways related to metabolites were represented via bubble plot

The top 10 significant DEMs identified in the POF vs CTRL group comprised those with upregulated expressions including 18-hydroxycorticosterone, 2-arachidonoylglycerol, rimantadine, pentostatin, mirtazapine, and taurocyamine and those with downregulated expressions including L-4-hydroxyphenylglycine, hydroquinone, retinol, and dicyclomine (Table 1). Additionally, these DEMs were verified via clustering heatmaps (Figure 4a) and further analyzed using Z-score (Figure 4b). The positive and negative correlations of these DEMs were determined using correlation coefficient analysis (Figure 4c). Moreover, the top 5 significantly enriched KEGG pathways for POF vs. CTRL group metabolites included ABC transporter-dependent pathways, protein digestion and absorption pathways, central carbon metabolism pathways in cancer, mineral absorption pathways, and cortisol synthesis and secretion pathways; these pathways were identified using the MetPA tool (Figure 4d and Table 2).

### PCOS vs POF metabolites

**Figure 5.**
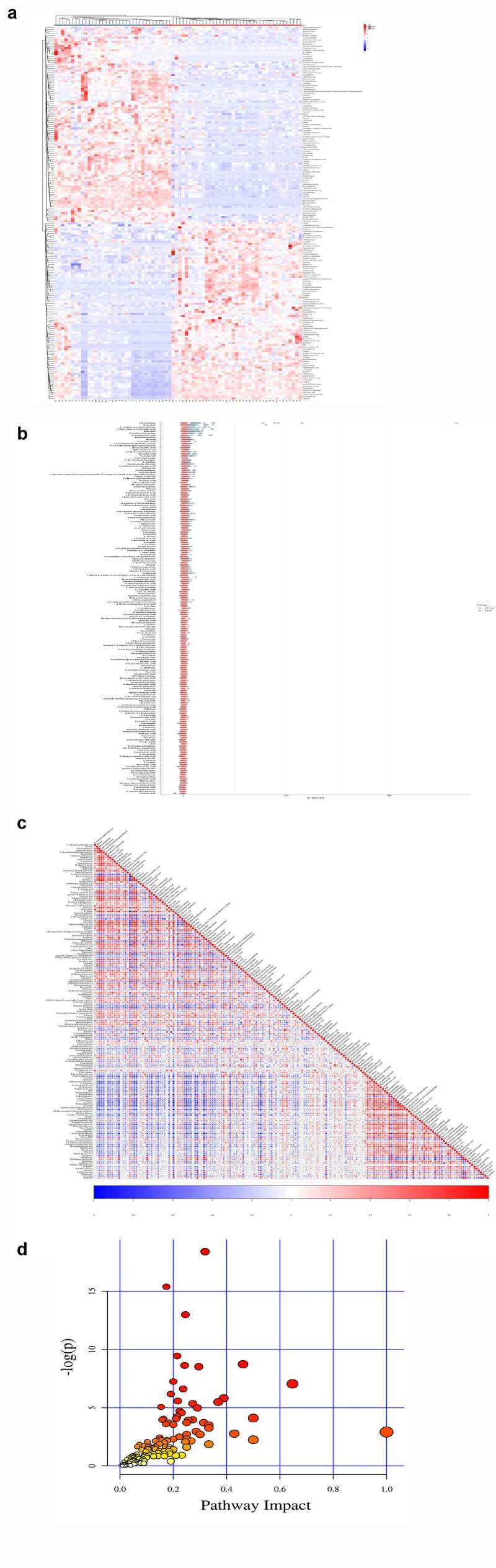
**The significant DEMs were determined in POF vs CTRL group**. (a) Heatmap clustering demonstrated the significant DEMs. (b) Z score statistical analysis was carried out for obtaining high-rank DEMs. (c) Pearson correlation coefficient analysis was performed between each DEM. (d) KEGG pathways related to metabolites were mentioned via bubble plot

The top 10 significant DEMs identified in the PCOS vs. POF group comprised those with upregulated expressions including L-4-hydroxyphenylglycine, dicyclomine, uracil-5-carboxylate, myo-inositol, and retinol and those with downregulated expressions including ethylmethylacetic acid, 2-archidonoylglycerol, D-fructose, 18-hydroxycorticosterone, and gamma-L-Glutamyl-L-2-aminobutyrate (Table 1). Furthermore, these DEMs were verified via clustering heatmaps and subsequently analyzed using Z-score. The positive and negative correlations of these DEMs were determined using correlation coefficient analysis (Figure 5(a-c)). In addition, the top 5 significantly enriched KEGG pathways for PCOS vs POF group metabolites included protein digestion and absorption pathways, ABC transporter-dependent pathways, central carbon metabolism pathways in cancer, aminoacyl-tRNA biosynthesis pathway, and prostate cancer pathways; these pathways were identified using the MetPA tool (Figure 5d and Table 2).

### Discovery of the most common DEMs among the three groups

**Figure 6.**
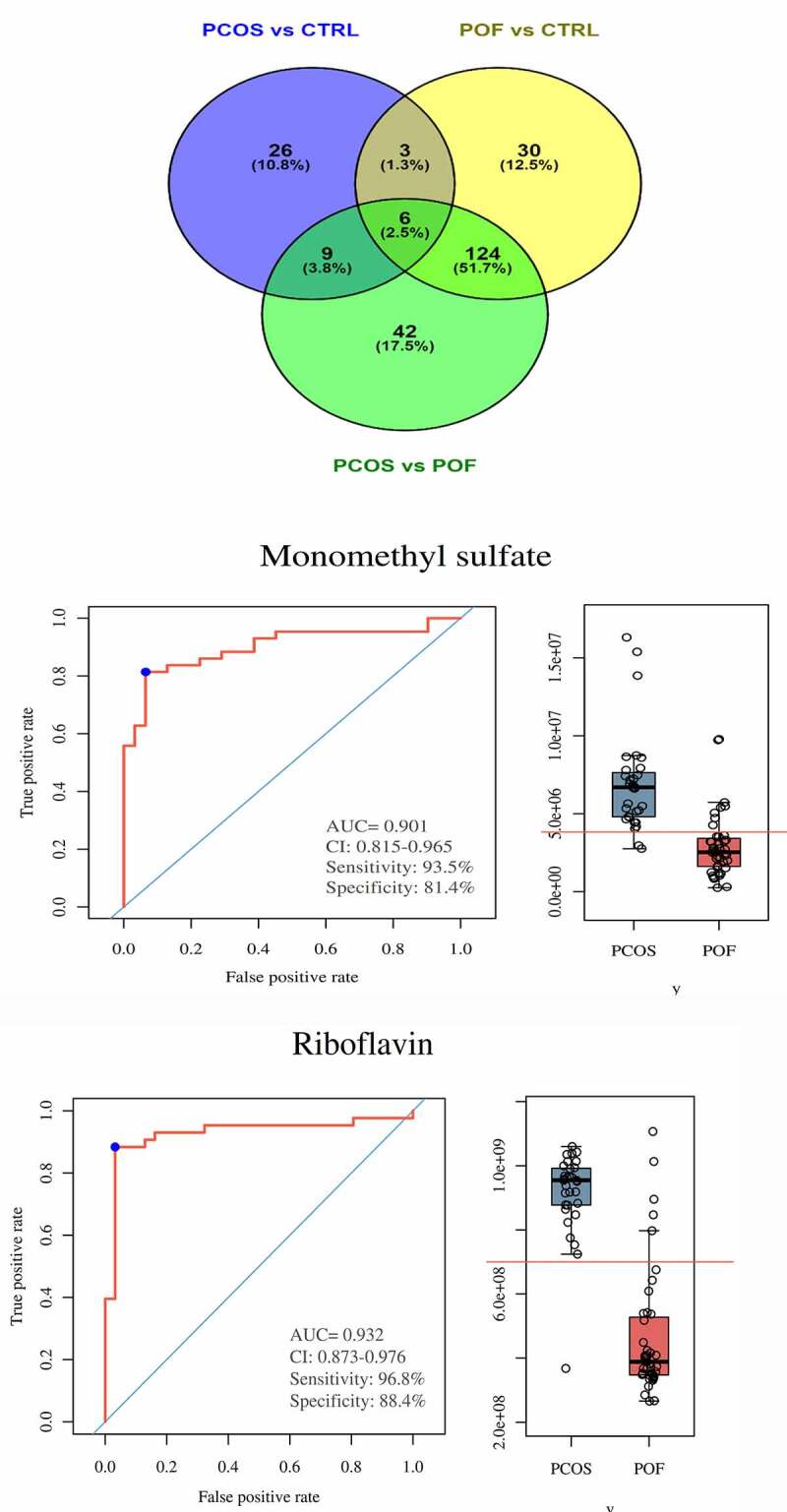
**Discovery and significance of most common metabolites in between three groups**. (a) Venn diagram demonstrated total and most commonly expressed DEMs in between comparison of three groups. (b,c) In PCOS vs POF group, Monomethyl sulfate and Riboflavin were highly expressed in PCOS compared to POF and represented good diagnostic efficiency by ROC-AUC (P < 0.05)

On the basis of threshold criteria, the top 44, 163, and 181 DEMs were identified in the comparisons of the PCOS vs CTRL, POF vs CTRL, and PCOS vs POF groups, respectively. Six DEMs were most frequently identified among the three groups using Venn diagram; these included monomethyl sulfate, riboflavin, oxoglutaric acid, 4-hydroxybenzoic acid, N-acetyldemethylphosphinothricin, and L-cysteine (Figure 6a). The intensity of each of these DEMs was measured across the three groups via bar plots (data not shown). The ROC of monomethyl sulfate yielded a significantly high AUC range of 0.77–0.957 (P < 0.05), with a sensitivity of 77.4%–90.7% and specificity of 76.9%–88.5%, whereas the ROC of riboflavin showed an AUC range of 0.702–0.932 (P < 0.05), with a sensitivity of 58.1%–96.8% and specificity of 84.5%–92.3% in the comparison among the three groups (Figure 6(b,c)**, Supplementary Figure S4 and Figure S5**).

**Figure 7.**
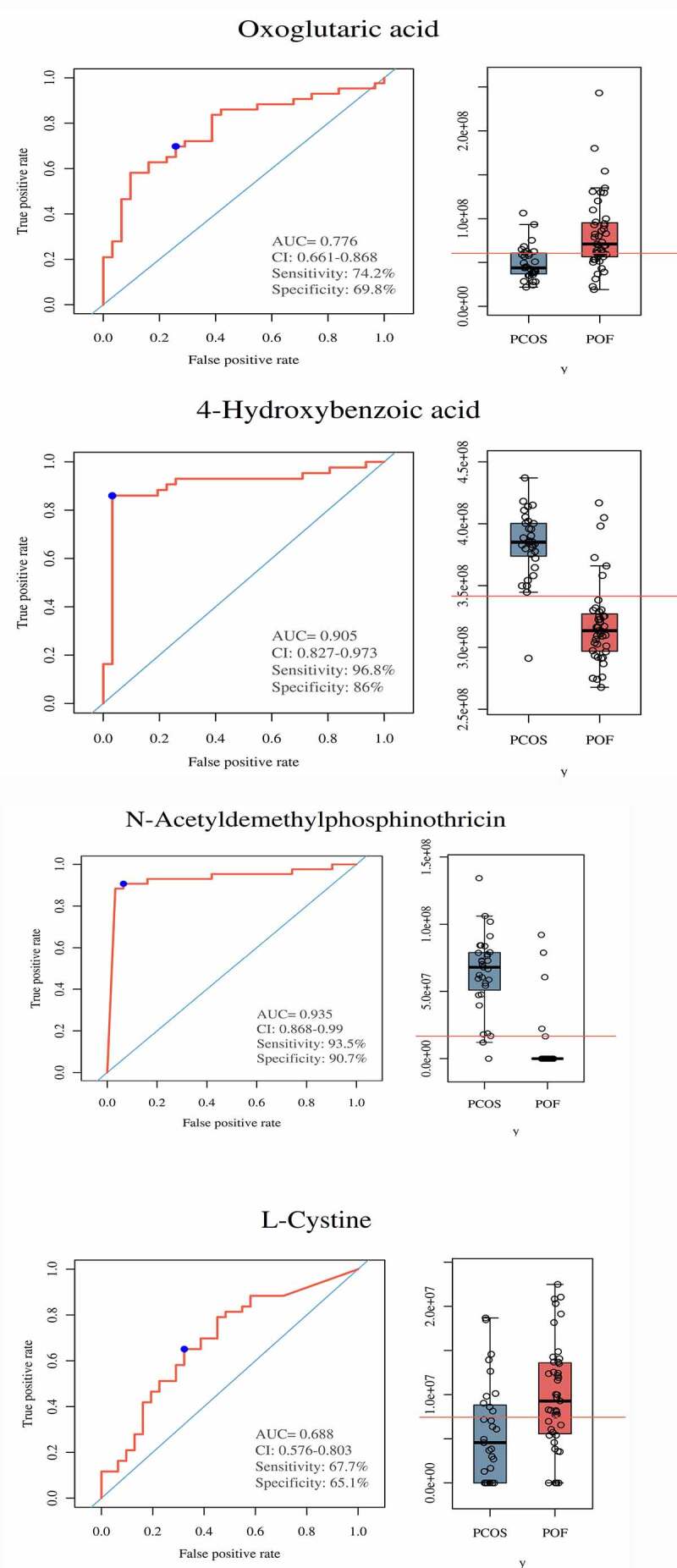
**Significance of metabolites in between PCOS and POF groups**. ROC curves and Bar plots showed clinical significance and expression of metabolites in a particular group. (a) Oxoglutaric acid, (b) 4-hydroxybenzoic acid, (c) N-Acetyldemethylphosphinothricin, (d) L-Cystine. They demonstrated promising diagnostic efficiency (P < 0.05)

The ROC of oxoglutaric acid yielded an AUC range of 0.676–0.878 (P < 0.05), with a sensitivity of 74.2%–83.7% and specificity of 61.5%–80.8%, whereas the ROC of 4-hydroxybenzoic acid yielded an AUC range of 0.658–0.905 (P < 0.05), with a sensitivity of 71%–96.8% and specificity of (57.7%–92.3%) (Figure 7(a,b)**, Supplementary Figure S4 and Figure S5**). The ROC of N-acetyldemethylphosphinothricin showed an AUC range of 0.663–0.935 (P < 0.05), with a sensitivity of 73.1%–93.5% and specificity of 61.5%–96.2%), whereas the ROC of L-cysteine yielded an AUC range of 0.678–0.828 (P < 0.05), with a sensitivity of 67.4%–80.6% and specificity of 61.5%–73.1% (Figure 7(c,d)**, Supplementary Figure S4 and Figure S5**). Monomethyl sulfate, riboflavin, 4-hydroxybenzoic acid, and N-acetyldemethylphosphinothricin expressions were most upregulated in the patients with PCOS and downregulated in those with POF. Furthermore, oxoglutaric acid and L-cysteine expressions were the most upregulated in the patients with POF and downregulated in those with PCOS. These results demonstrate the efficacy of these 6 metabolites in accurately distinguishing between patients with PCOS and POF using serum samples, showing the significant potential of these metabolites in the diagnosis of PCOS and POF.

## Discussion

PCOS is a complex endocrinopathy and a leading cause of infertility due to anovulation or oligoovulation [20]. Conversely, POF is a highly heterogeneous disorder and mostly occurs after treatments for autoimmune and neoplastic diseases [21]. Despite the soaring incidence of these disorders, their underlying pathophysiological mechanisms remain unclear [20,21]. Thus, the treatment of PCOS or POF is multidimensional and takes into account aspects such as genetics, symptoms of infertility and hyperandrogenism, insulin resistance, and their metabolic reactions [22]. Therefore, it is crucial to ascertain the underlying pathophysiological mechanisms of these syndromes by determining significant biomarkers using noninvasive, next-generation technology-based methods. Currently, the application of metabolomics, which is an emerging but powerful tool, represents one such method [23,24].

The discovery of novel significant serum biomarkers for the screening and diagnosis of PCOS and POF, especially in early stages, has recently become a critical goal. Nevertheless, not many biomarker candidates have been clinically applied because of inadequate study cohorts/participants or diagnostic efficacies. In our study, a total of 100 participants constituting the PCOS, POF, and healthy CTRL groups were enrolled from a single center. We used LC–MS-based metabolomics to identify the biomarkers. Few undiscovered metabolites might act as biomarkers; therefore, these metabolites need to be determined. In the present study, we focused on discovering numerous metabolites. On the basis of threshold criteria and univariate and multivariate analysis results, six biomarkers including monomethyl sulfate, riboflavin, oxoglutaric acid, 4-hydroxybenzoic acid, N-acetyldemethylphosphinothricin, and L-cysteine were discovered and their up/downregulated expressions verified in the PCOS, POF, and CTRL groups. Furthermore, these serum-based metabolic biomarkers could significantly distinguish between PCOS and POF with an AUC of 0.65–0.95, at a sensitivity of 58.8%–96.8% and specificity of 57.9%–96.2%. These six novel biomarkers exhibited high diagnostic efficacy and accuracy and marked complementarity to differentiate between PCOS and POF.

PCOS and POF are accompanied by various systemic metabolic alterations, such as dysregulated glucose metabolism and insulin resistance, that might affect ovarian follicles; furthermore, these metabolic abnormalities might lead to alterations in the composition of body fluids such as follicular fluid and serum or plasma [25,26]. Reportedly, dysregulated glucose metabolism and insulin resistance affect multiple energy pathways, manifesting as altered follicular fluid concentrations of different biomolecules such as amino acids, lipids, and ketone bodies [27,28]. Moreover, concentrations of certain free fatty acids in the follicular fluid and serum are altered in patients with PCOS [29,30]. Studies have reported that insufficient availability of methyl groups may induce critical hypothalamic–pituitary–ovarian axis-related gene regulatory mechanisms implicated in PCOS progression, and metabolism regulates methyl group transfer, which is critical for homocysteine homeostasis [31,32] However, homocysteinemia is positively associated with PCOS and other diseases [33]. The imbalance in methyl group metabolism could be the main pathophysiological mechanism underlying the occurrence and progression of PCOS. Patients with POF exhibit high homocysteine concentrations, which are, in turn, related to elevated follicle-stimulating hormone and low serum estradiol levels [34]. L-cysteine, N-acetyldemethylphosphinothricin, and oxoglutaric acid, discovered as biomarkers in the present study, are types of amino acids that are involved in the metabolic pathways of amino acids [35–37]. Women with PCOS are deficient in riboflavin (vitamin B_2_); furthermore, vitamins (water-soluble) play important roles in the therapy of women with PCOS and POF by reducing the antioxidative stress and low-intensity inflammation caused by several factors, in addition to chronic infection [38]. Monomethyl sulfate and 4-hydroxybenzoic acid are chemicals that may act as metabolites [37,39]. In the present study, we discovered six metabolites with up/downregulated expressions in serum samples from the PCOS, POF, and healthy CTRL groups, similar to previous metabolomics and proteomics studies identifying novel biomarkers for PCOS, POF, and other neoplastic diseases [18,19,25,40–44]. Taken together, these 6 biomarkers may not only affect the pathogenesis of PCOS/POF but also accurately differentiate patients with PCOS or POF from healthy individuals. Therefore, their clinical application can be considered after validation in larger cohorts, which may also guide future studies on this subject.

However, this study has few limitations. First, the metabolic profiling of the discovered metabolites was performed in a single center-based cohort, which might represent biased samples or findings. Second, the validation of these common metabolites was not performed; therefore, multicenter studies with a larger cohort are warranted for validating these metabolites. Third, the six different metabolites were evaluated and compared using only serum samples, and plasma or other body fluid-based samples were not used. Therefore, further studies are needed to detect these metabolites in other fluid samples and evaluate their association with various corresponding genes, which may play a role in the occurrence and progression of PCOS and POF.

## Conclusion

Novel metabolites with up/downregulated expressions can be discovered in patients with PCOS and POF using serum-based metabolomics; these metabolites show good diagnostic performance and can act as effective biomarkers for the early detection of PCOS and POF. Furthermore, these metabolites might be involved in the pathophysiological mechanisms underlying the occurrence and progression of PCOS and POF via interplay with corresponding genes.

## Supplementary Material

Supplemental Material

## Research highlights

1. Discovered six novel metabolites in patients with PCOS and POF

2. Representing good diagnostic performances as serum metabolites

3. May take part in the pathophysiological mechanism of PCOS and POF
