# Supplementary material for: Discovery of novel serum metabolic biomarkers in patients with polycystic ovarian syndrome and premature ovarian failure
Source: Bioengineered. 2021 Oct 26;12(1):8778–92. doi: 10.1080/21655979.2021.1982312 (PMC8806610; doi:10.1080/21655979.2021.1982312)
Supplement: Supplemental Material [file KBIE_A_1982312_SM7415.docx]

**Supplementary Figures**

**A**


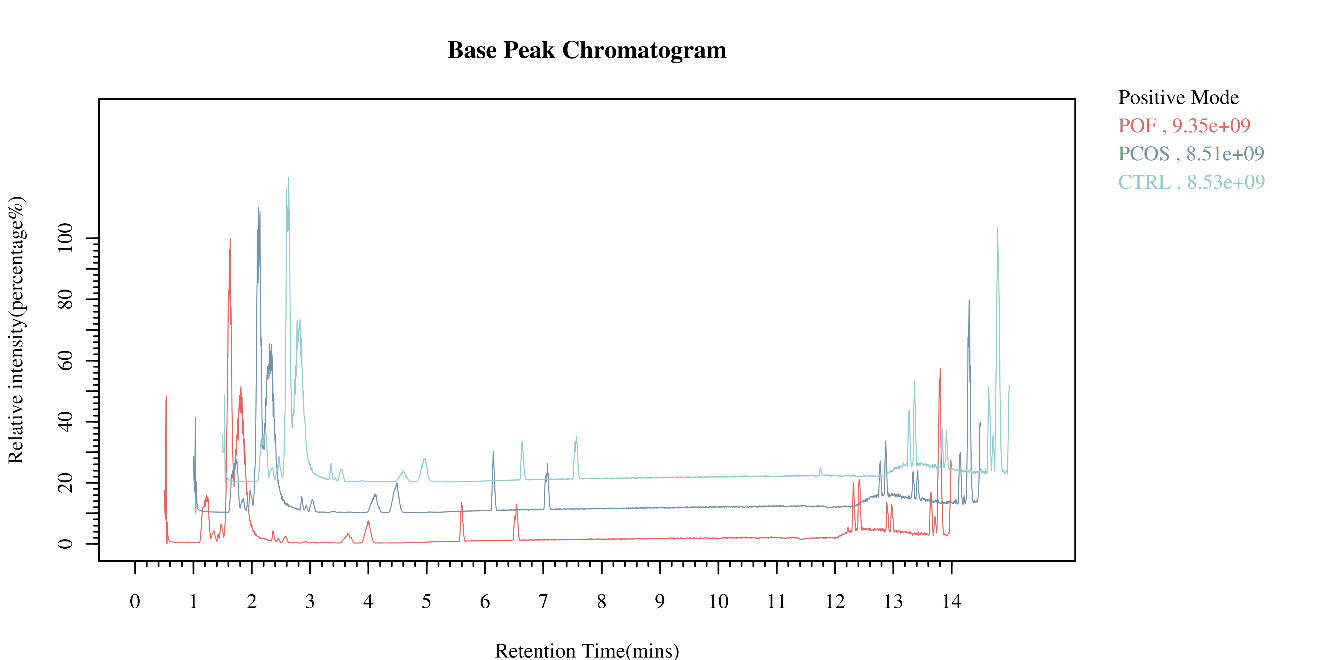


**B**


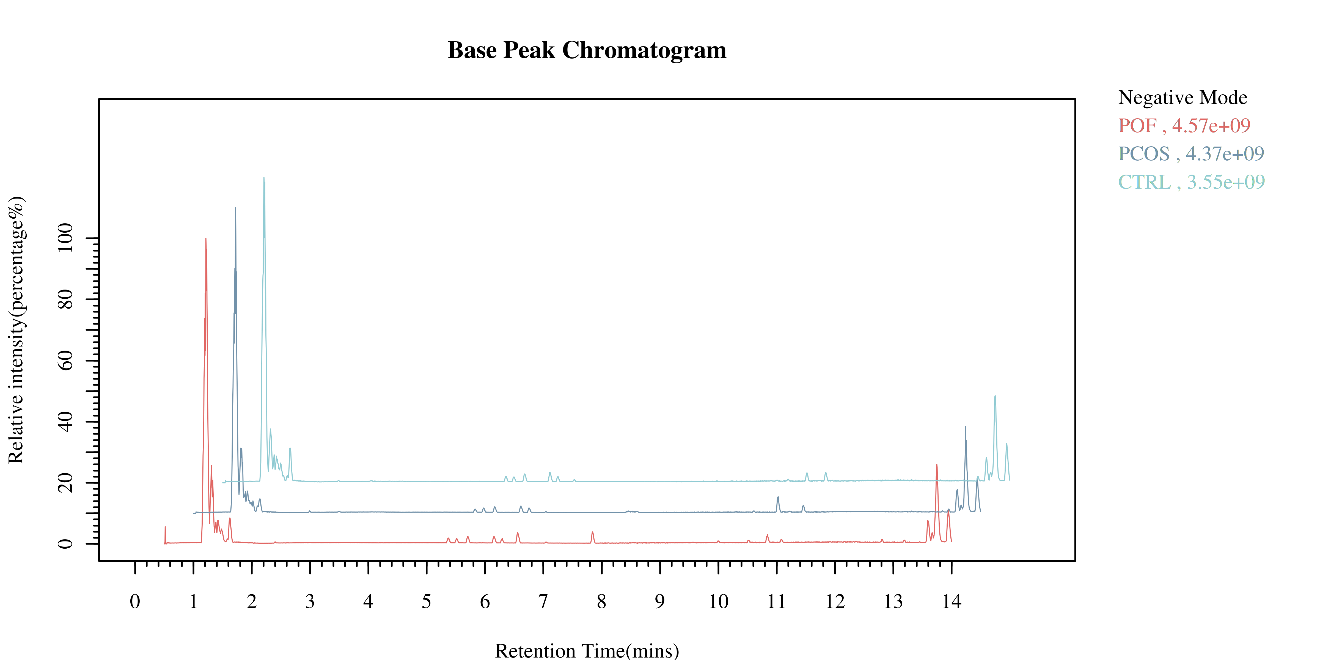


**Figure S1. Base peak chromatogram analyzed the ion ESI+ and ESI- specific intensity of three groups**. (ESI^+^), and ESI^-^(negative modes)

**A C**


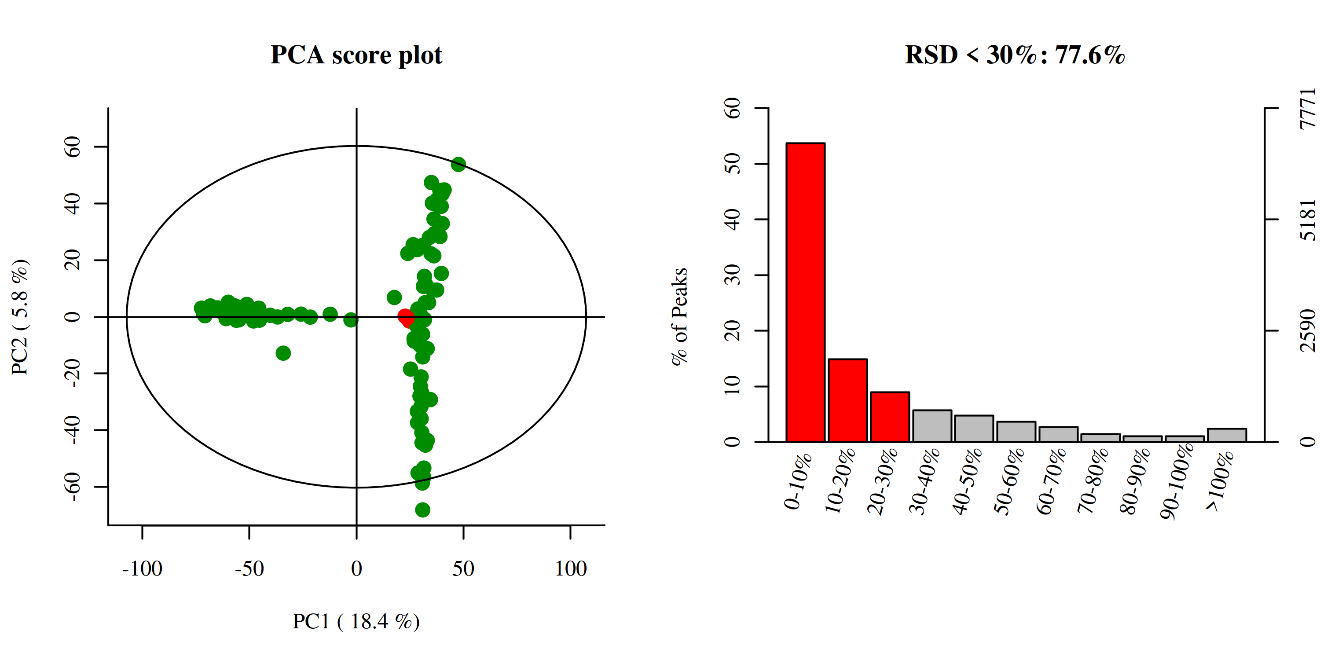


**B D**


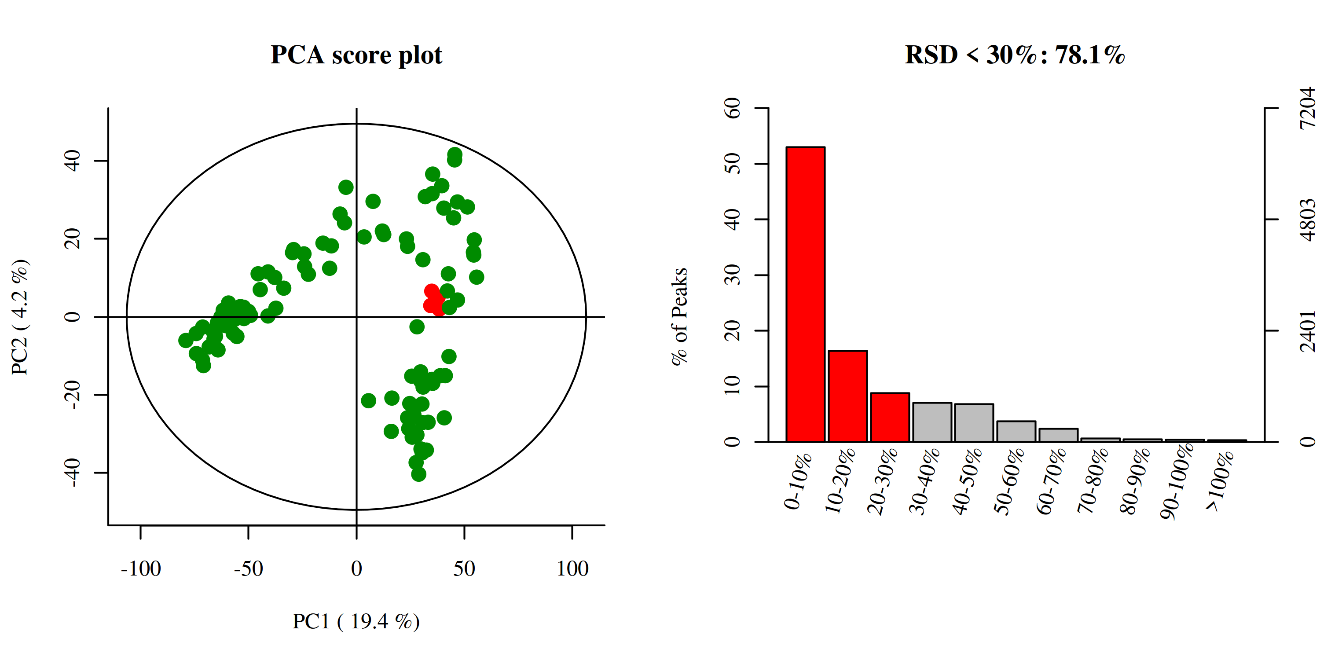


**Figure S2. Quality check and quality assurance were assessed before metabolomics.** (A,B) Principal component analysis of positive and negative modes. (C,D) Relative standard deviation tops at a coefficient of variation by histogram.

**A B**


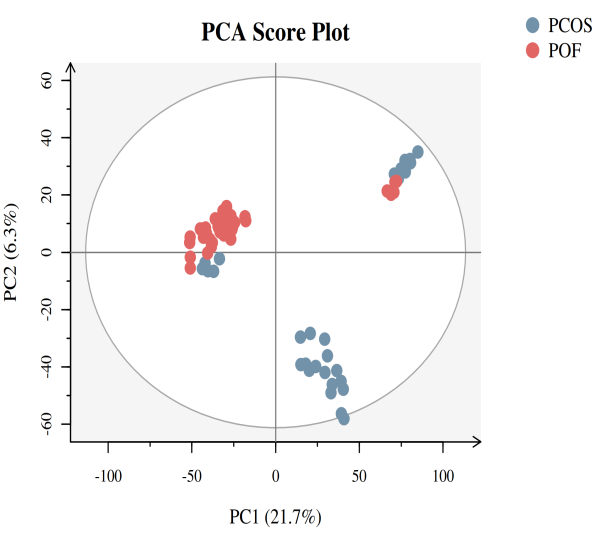

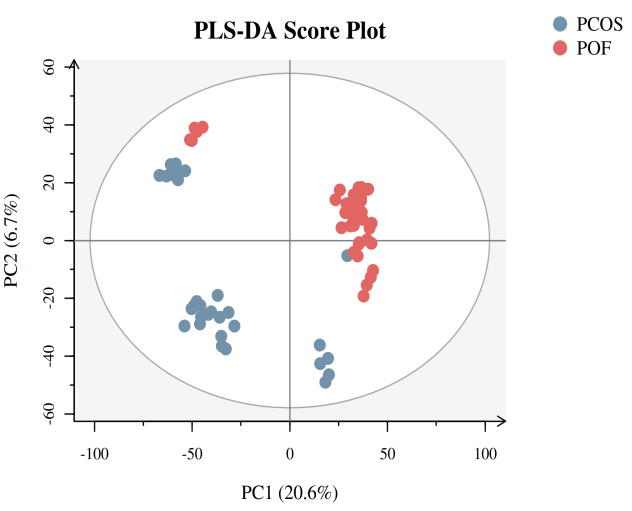


**C D**


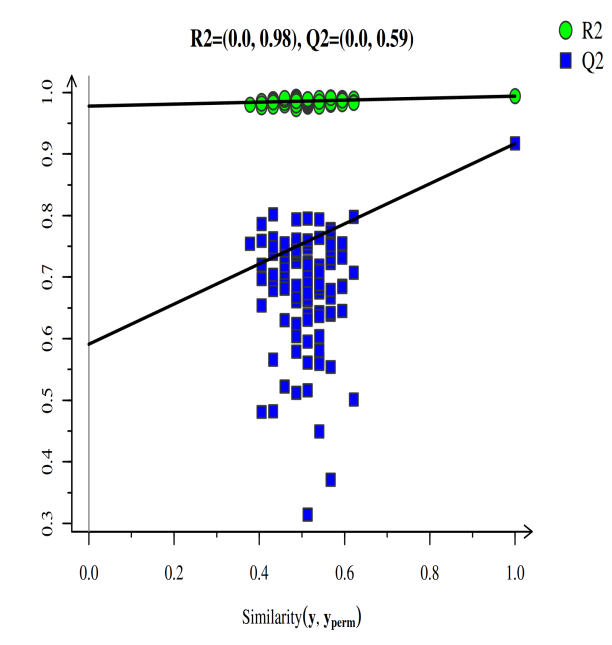

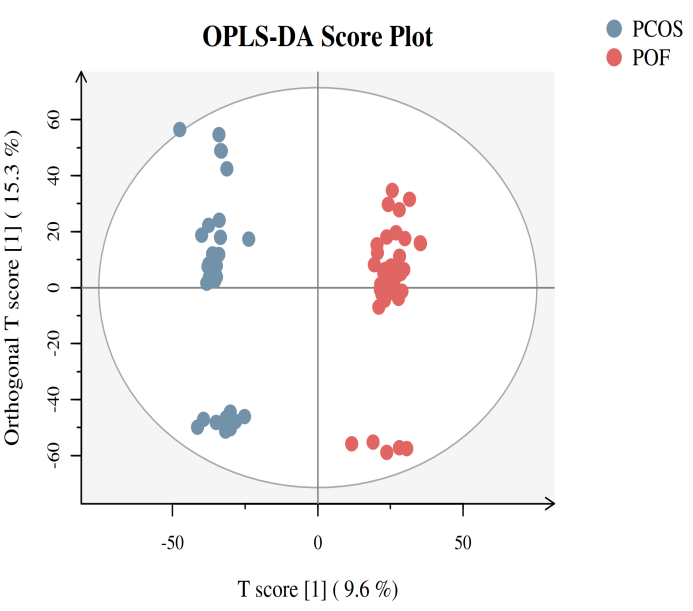


**Figure S3. Score plots of three different analyses based on ESI- mode in PCOS and POF.** (A) Principal component analysis (PCA), (B,C) Partial least discriminant analysis (PLS-DA) (D) Orthogonal partial least square discriminant analysis (OPLS-DA) was used for displaying PCOS and POF subjects.


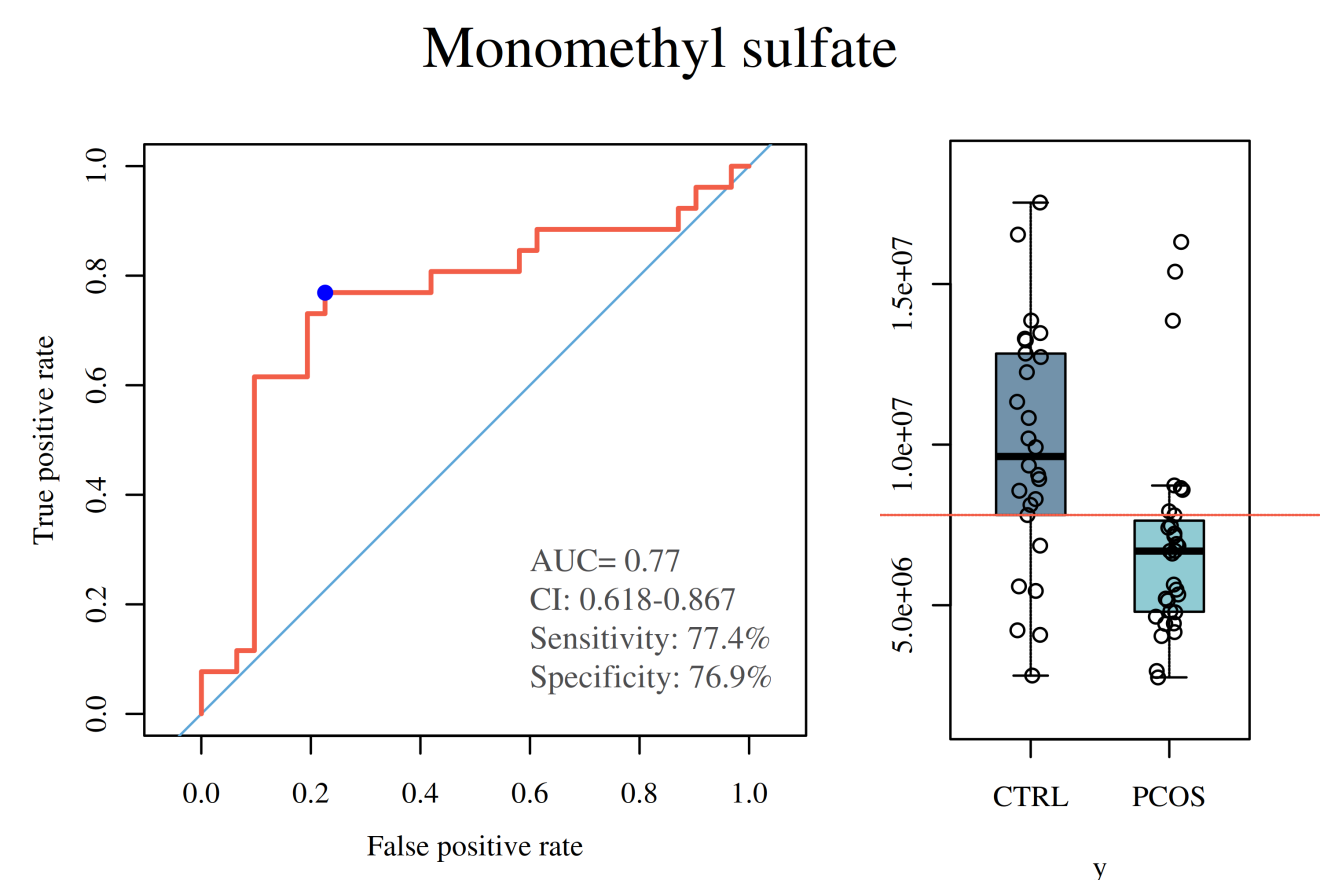

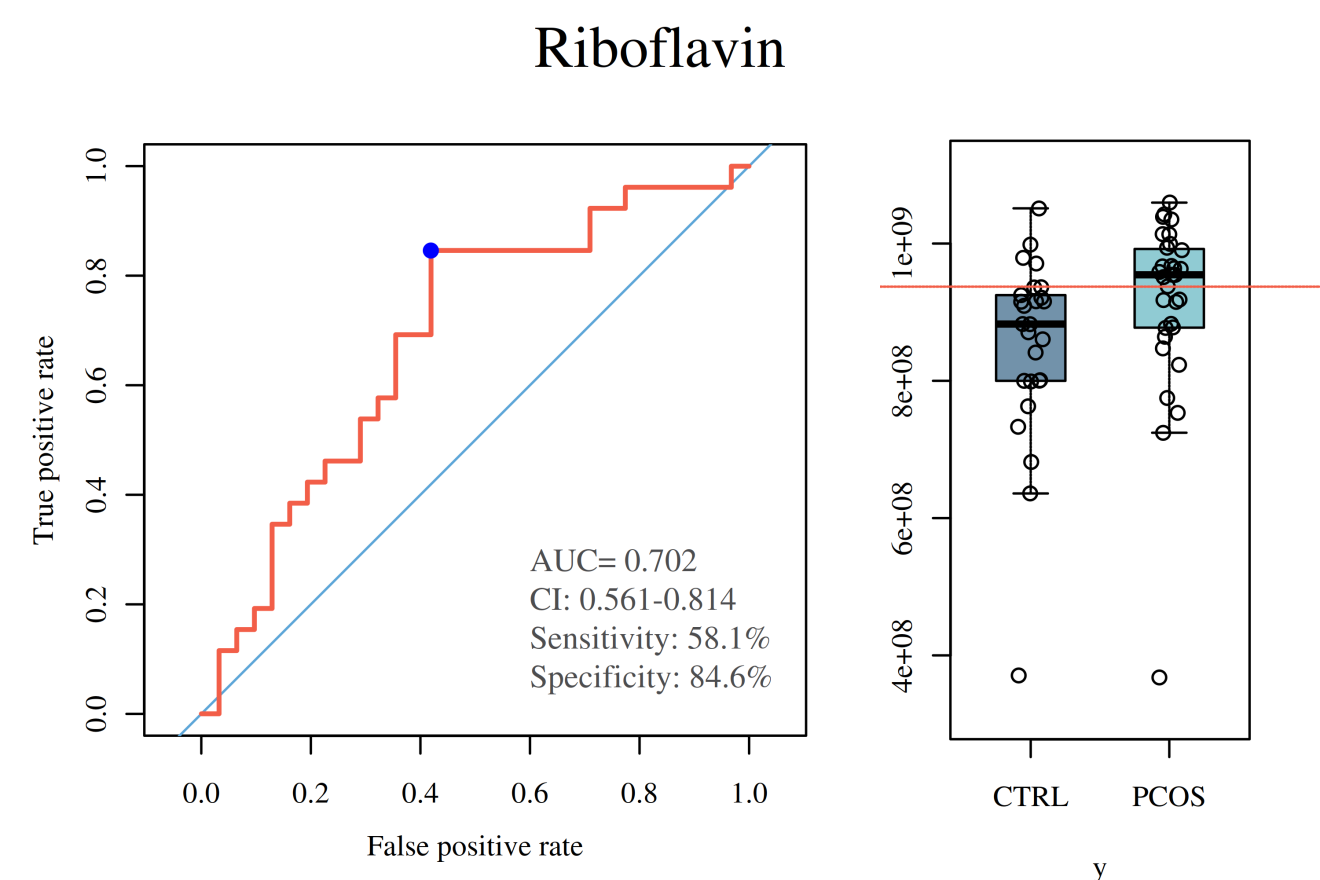

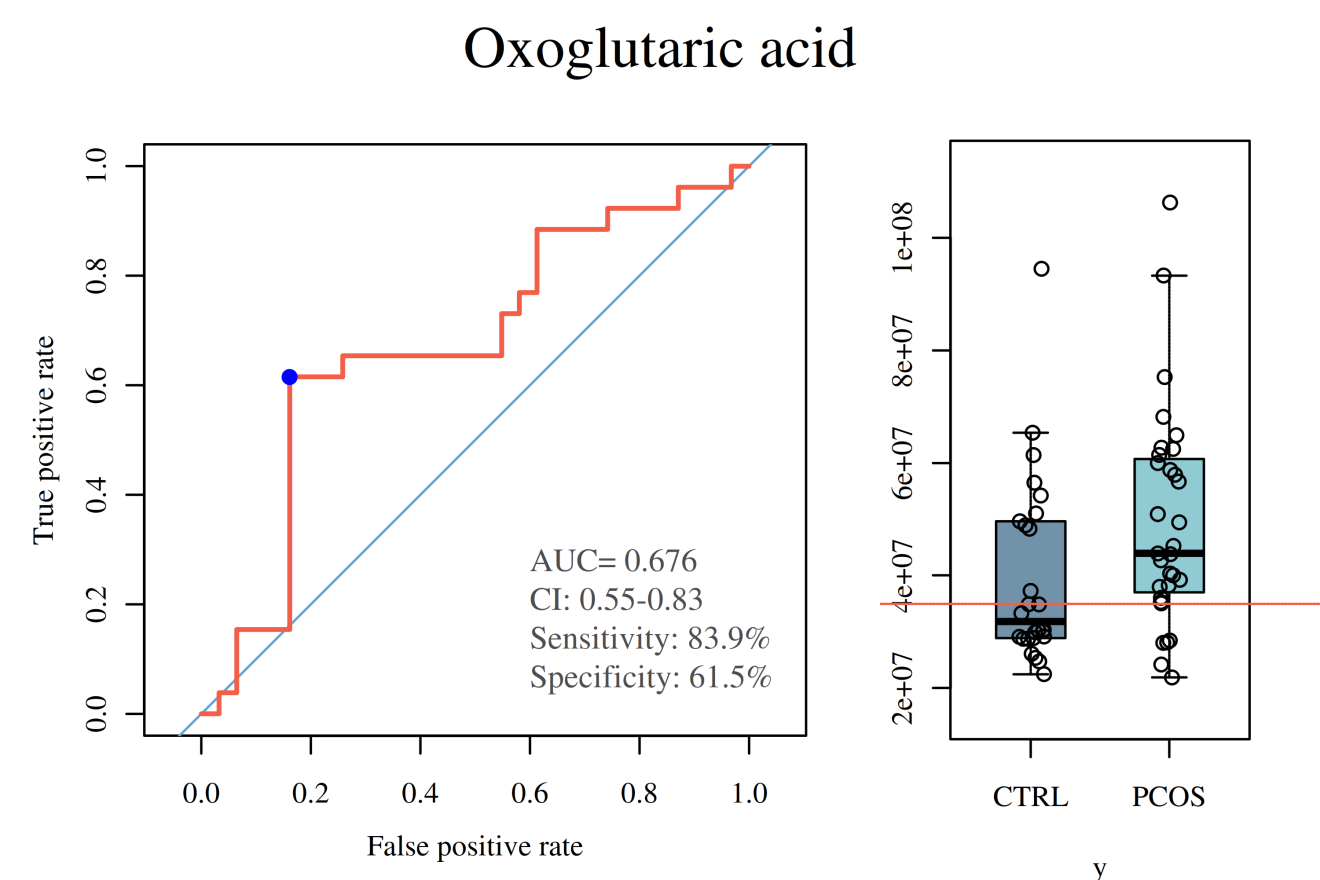

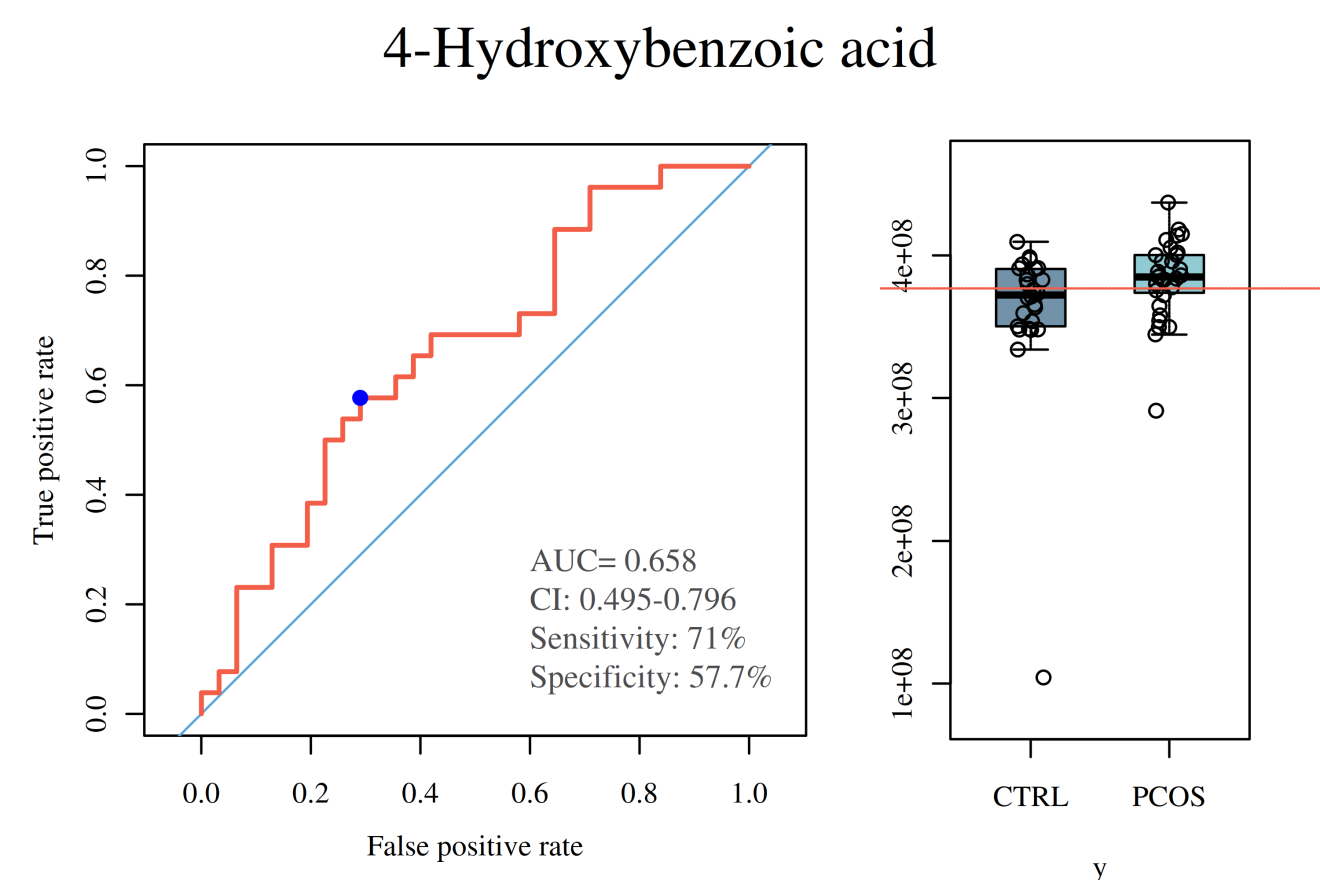

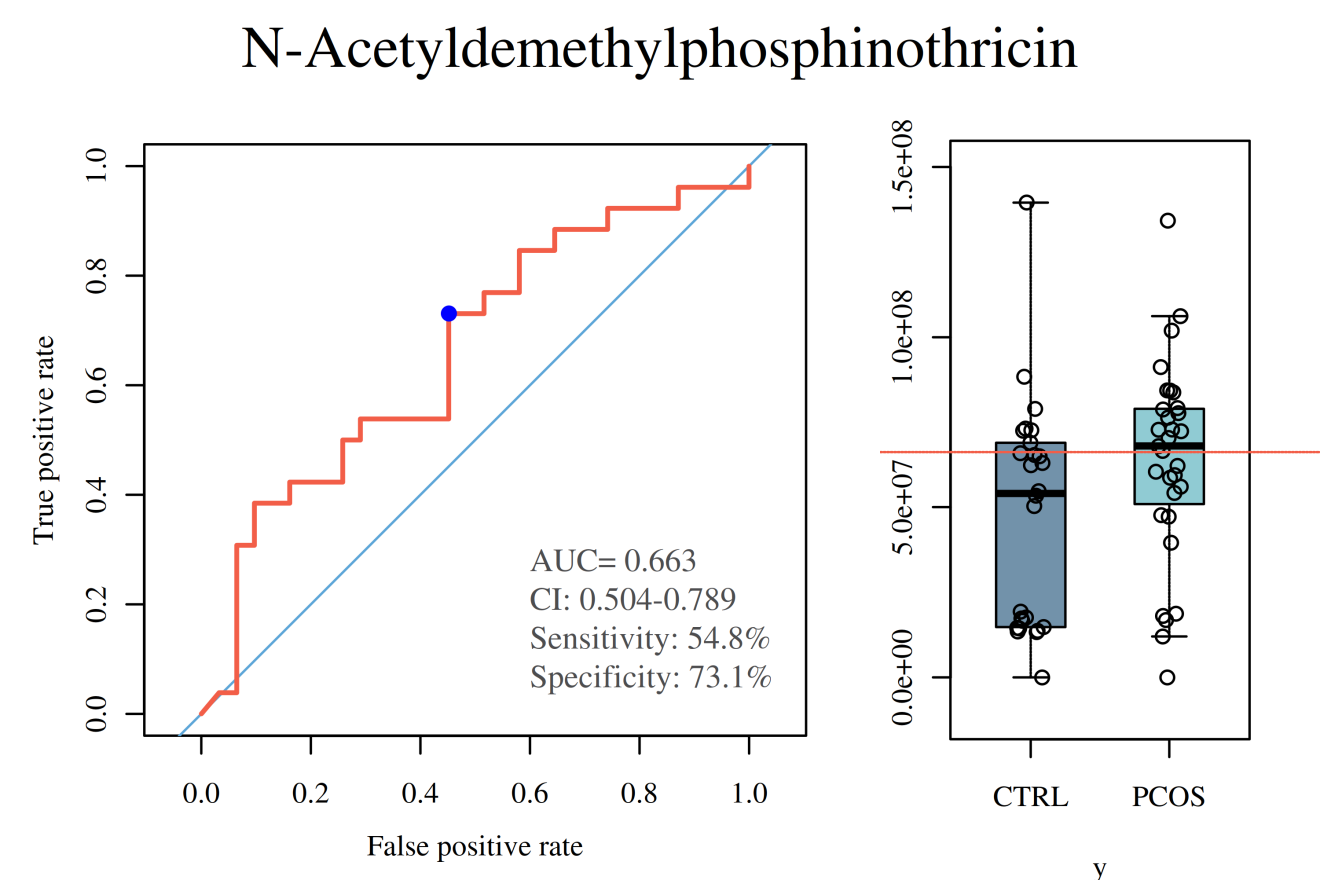

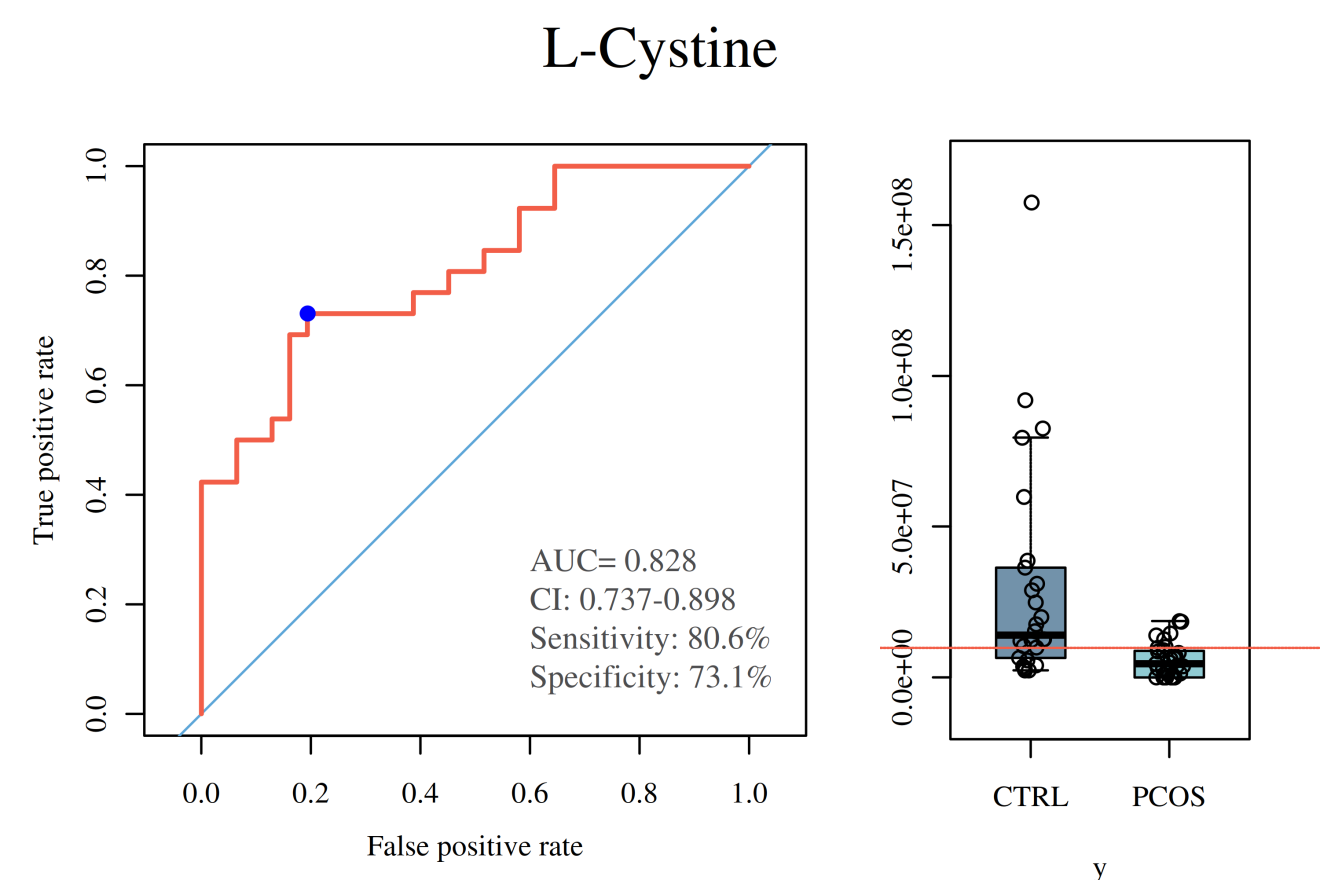


**Figure S4. ROC curves of six metabolites in PCOS vs CTRL group.**


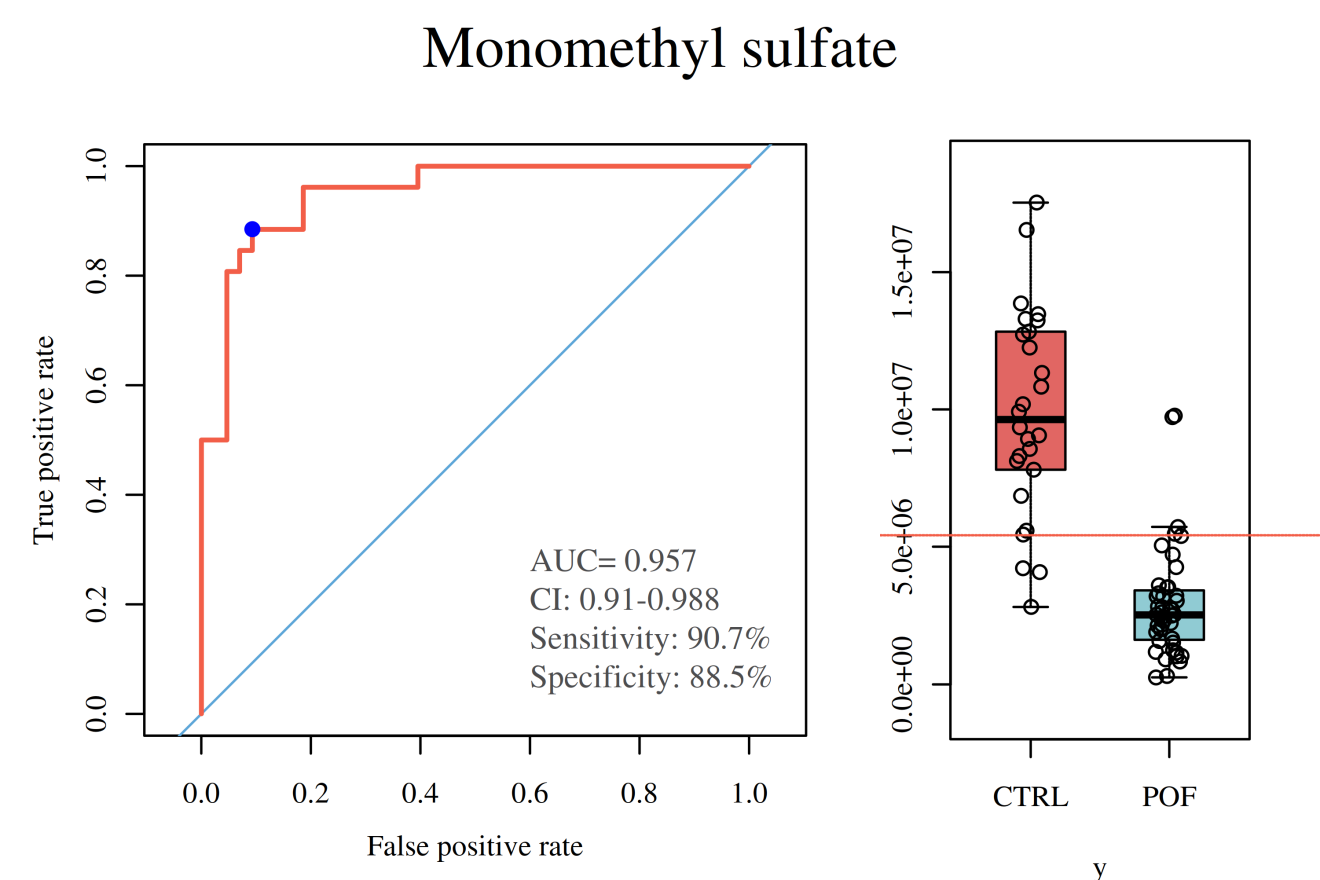

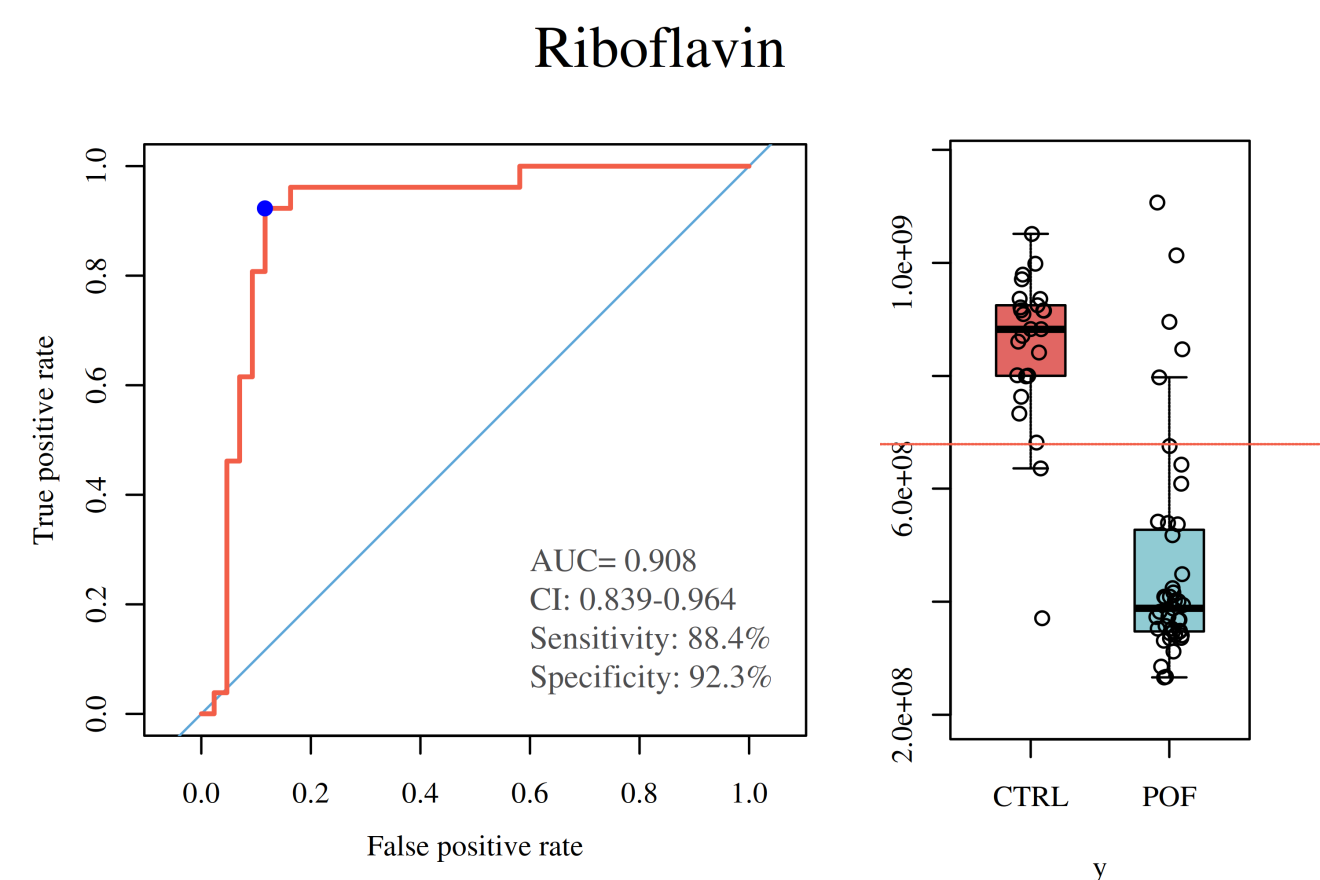

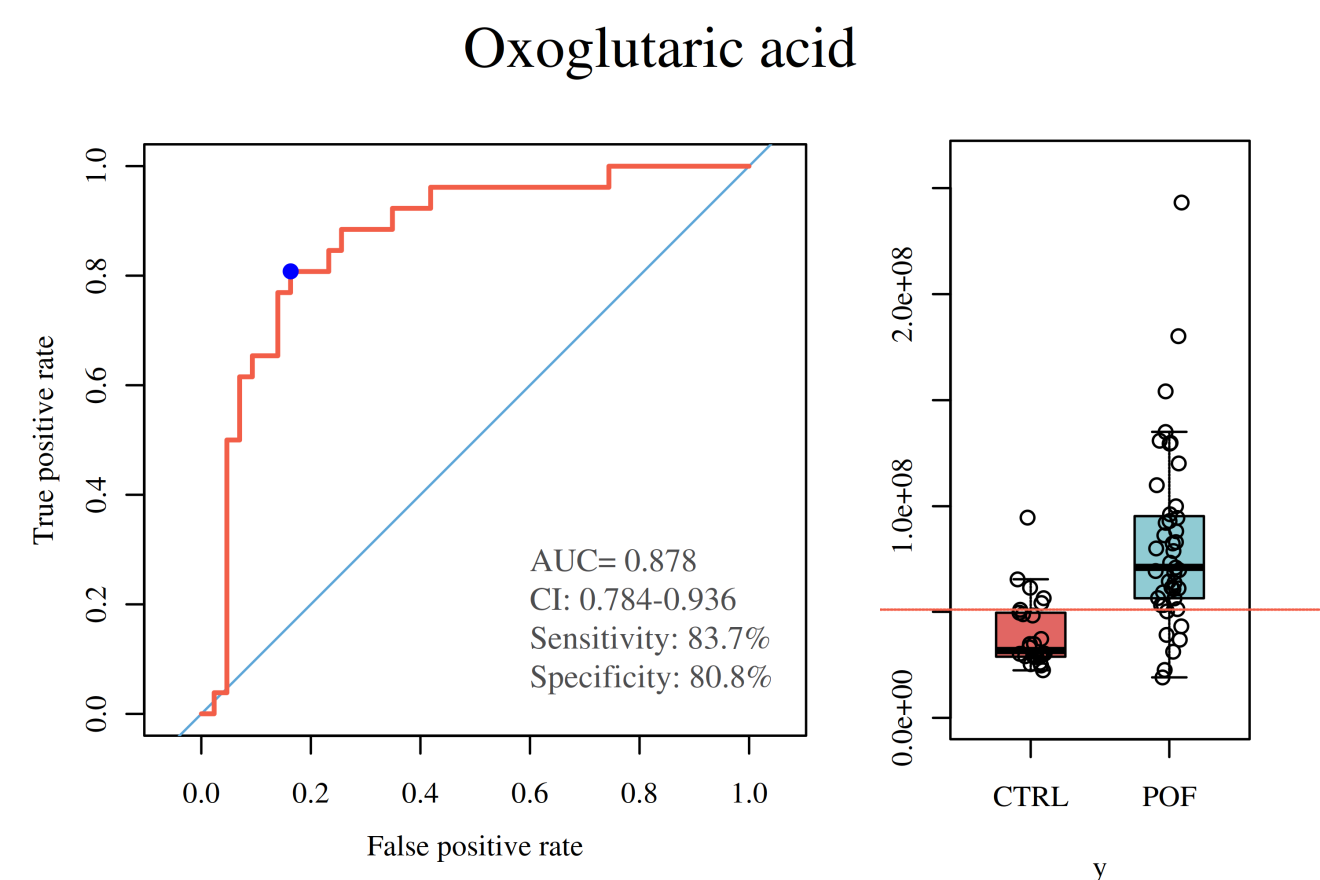

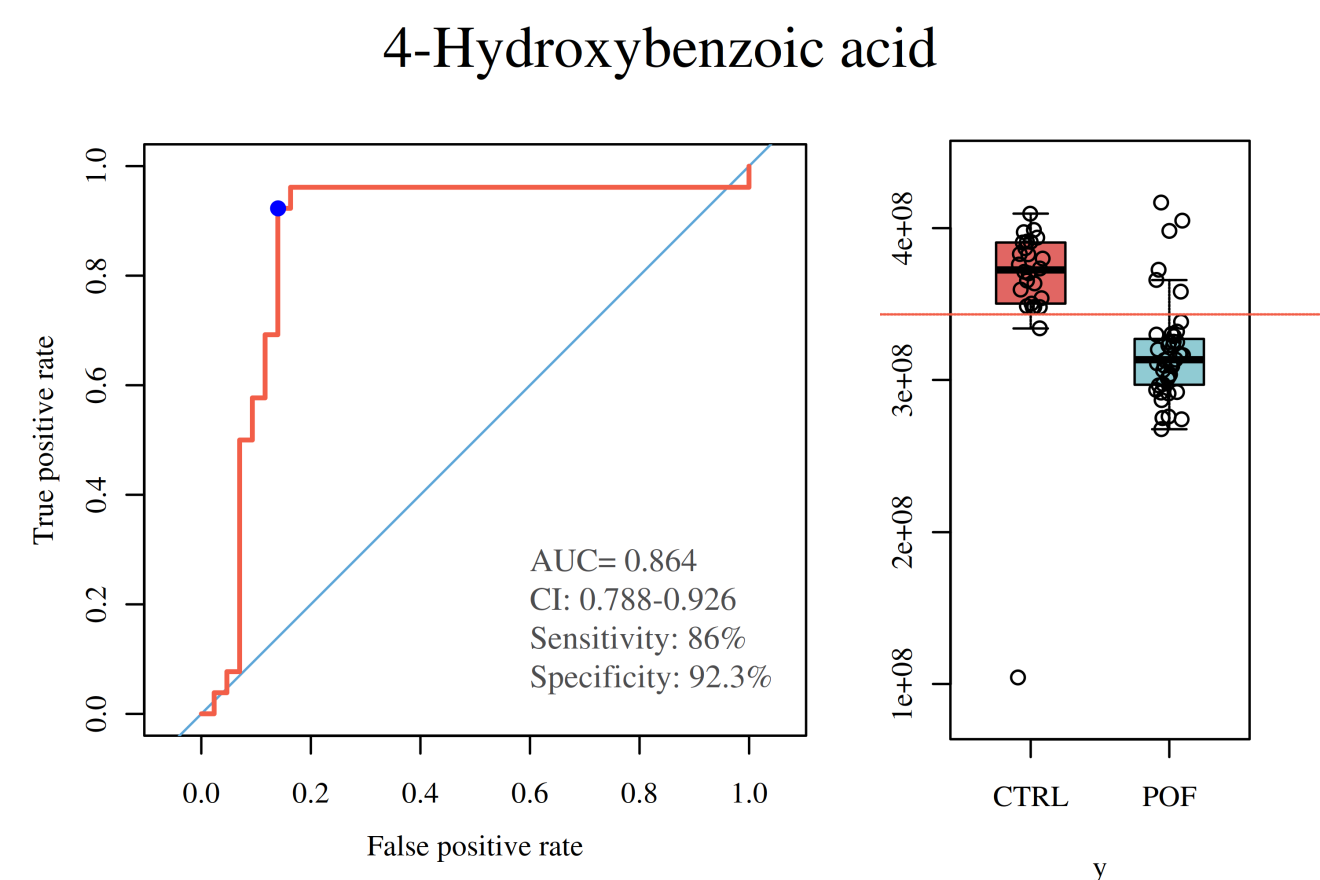

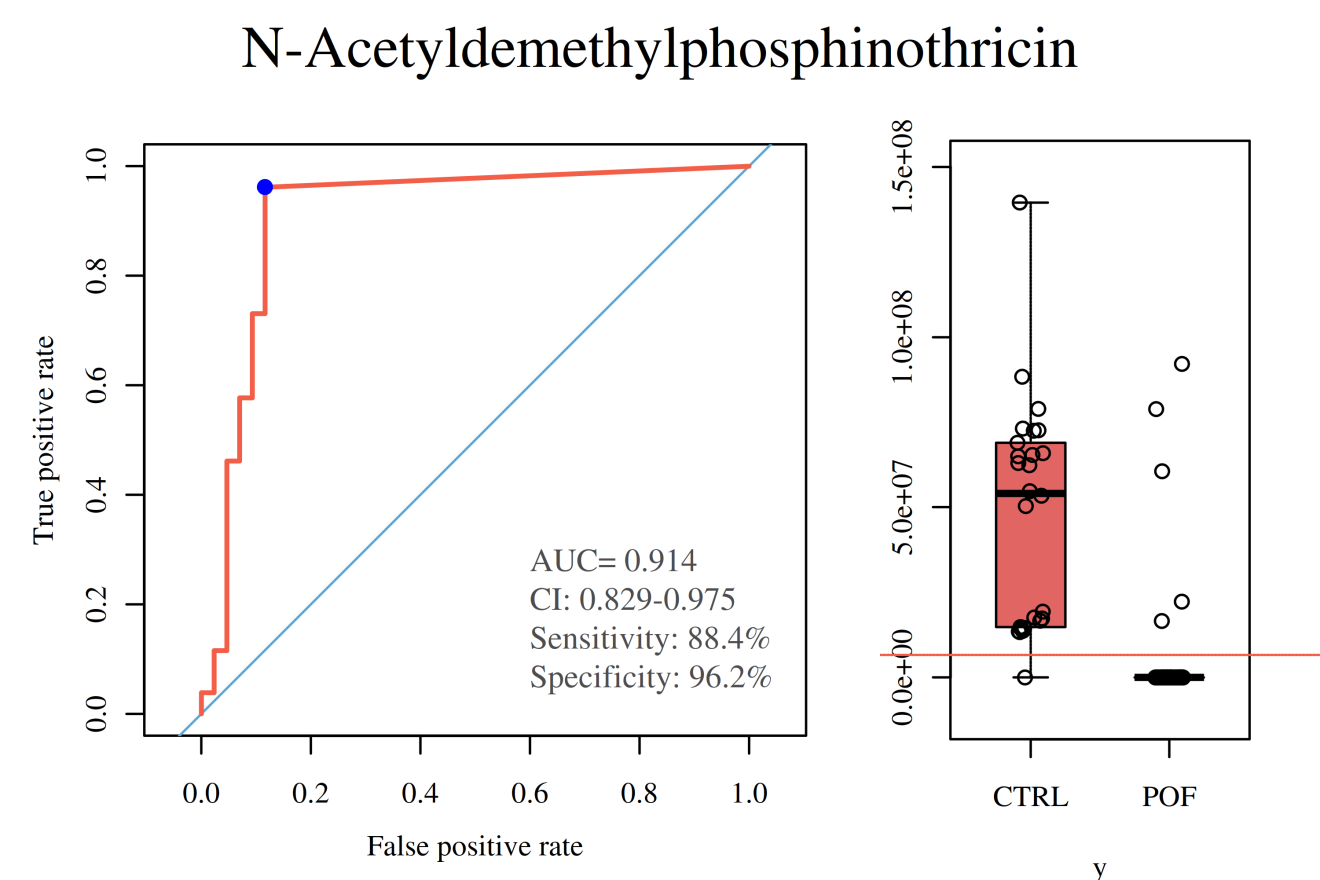

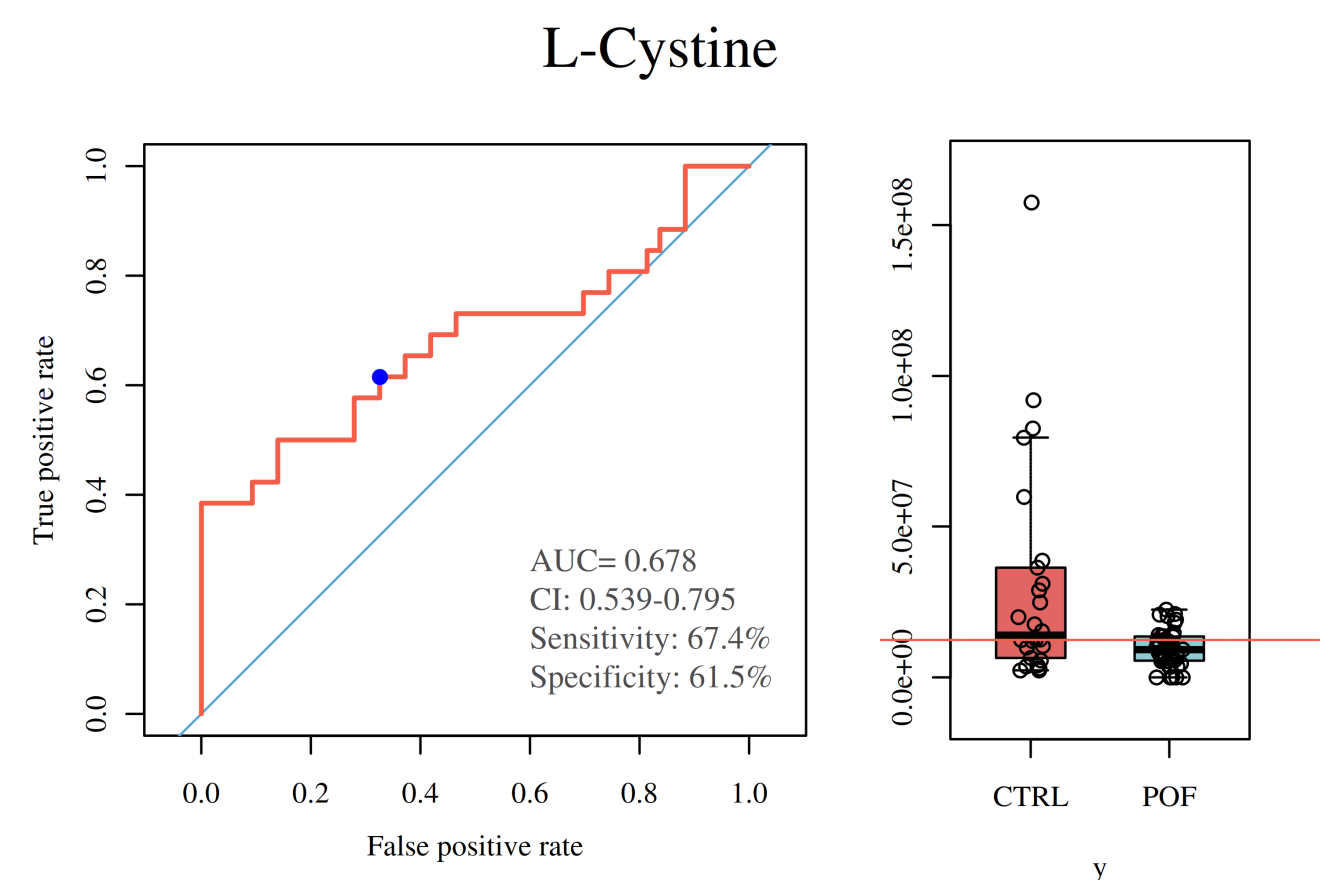


**Figure S5. ROC curves of six metabolites in POF vs CTRL group.**
